# HGF/c-Met pathway inhibition combined with chemotherapy increases cytotoxic T-cell infiltration and inhibits pancreatic tumour growth and metastasis

**DOI:** 10.1016/j.canlet.2023.216286

**Published:** 2023-06-22

**Authors:** Alpha Raj Mekapogu, Zhihong Xu, Srinivasa Pothula, Chamini Perera, Tony Pang, S.M. Zahid Hosen, Vishnu Damalanka, James Janetka, David Goldstein, Romano Pirola, Jeremy Wilson, Minoti Apte

**Affiliations:** a Pancreatic Research Group, South West Sydney Clinical Campuses, School of Clinical Medicine, Faculty of Medicine and Health, UNSW Sydney, Australia; b Ingham Institute for Applied Medical Research, Sydney, Australia; c AbCellera, Beaconsfield, New South Wales, United Kingdom; d Surgical Innovations Unit, Westmead Hospital, Sydney, Australia; e Westmead Clinical School, Faculty of Medicine and Health, The University of Sydney, Sydney, Australia; f Department of Biochemistry and Molecular Biophysics, Washington University, St. Louis, USA

**Keywords:** Pancreatic neoplasm, Fibroblast, Mesenchymal-epithelial transition MET, Apoptosis, Macrophage

## Abstract

Pancreatic cancer (PC) is a deadly cancer with a high mortality rate. The unique characteristics of PC, including desmoplasia and immunosuppression, have made it difficult to develop effective treatment strategies. Pancreatic stellate cells (PSCs) play a crucial role in the progression of the disease by interacting with cancer cells. One of the key mediators of PSC - cancer cell interactions is the hepatocyte growth factor (HGF)/c-MET pathway. Using an immunocompetent *in vivo* model of PC as well as in vitro experiments, this study has shown that a combined approach using HGF/c-MET inhibitors to target stromal-tumour interactions and chemotherapy (gemcitabine) to target cancer cells effectively decreases tumour volume, EMT, and stemness, and importantly, eliminates metastasis. Notably, HGF/c-MET inhibition decreases TGF-β secretion by cancer cells, resulting in an increase in cytotoxic T-cell infiltration, thus contributing to cancer cell death in tumours. HGF/c-MET inhibition + chemotherapy was also found to normalise the gut microbiome and improve gut microbial diversity. These findings provide a strong platform for assessment of this triple therapy (HGF/c-MET inhibition + chemotherapy) approach in the clinical setting.

## 1. Introduction

Pancreatic cancer (PC) is characterised by local and systemic immunosuppression [1] and extensive desmoplasia/stroma, which constitutes more than 70% of the tumour volume [2]. The five-year survival rate of PC is dismal, ranging from 2% to 11% globally [3–6] and 12% in Australia [7]. Therapeutic options for PC are limited due to late diagnosis and ineffective therapies. Surgical resection combined with chemotherapy is the only curative option [8,9], but only 10% of patients are diagnosed at a resectable stage, while the remaining 90% remain unresectable with 30% having locally advanced tumours and 60% having metastatic disease [10]. Although recent therapeutic combinations, such as FOLFIRINOX and gemcitabine + nab-paclitaxel, have marginally improved the survival of PC patients with advanced disease [8,11], their use is limited due to substantial side effects [12,13]. Thus, there is an unmet need for safe and novel therapeutics for treating advanced/unresectable PC.

Pancreatic stellate cells (PSCs) comprise 4–7% [14] of the total parenchyma and are located around the basolateral aspects of the acinar cells. PSCs in their native state express abundant vitamin A (retinoids) containing droplets in their cytoplasm and express stellate cell selective markers such as desmin, glial fibrillary acidic protein (GFAP), nestin, neural cell adhesion molecule, nerve growth factor, fatty acid binding protein 4 (Fabp4) and synemin [15–17]. In PC, activated PSCs are the primary source of the collagenous stroma [18]. Activated PSCs play a major role in the production of collagenous stroma in PC, and also interact closely with cancer cells to promote cancer progression. Among pathways that mediate PSC and cancer cell interactions, the HGF/c-MET pathway is interesting for two reasons: (i) HGF and or c-MET expression has been reported to be upregulated in many cancers including PC and this is often associated with a poor prognosis [19,20]. (ii) this pathway is uniquely compartmentalized between PSC and cancer cells. The ligand HGF is secreted by PSCs, while the receptor c-MET is present in pancreatic cancer cells [21]. Targeting this pathway has been shown to decrease tumour growth and eliminate metastasis in immunodeficient xenograft models [22,23]. However, these studies are limited by the absence of a competent immune system and given the key role of immune cells in cancer progression, it is critical to assess interventions in the presence of an active host immune system.

PC is also associated with dysbiosis of the gut microbiota characterised by decreased microbial diversity and increased pathogenic organisms. These changes are linked to the progression of PC and poor long-term survival of PC patients [24]. More recently, the gut microbiota has been shown to play a role in suppressing antitumour immunity and causing resistance to anticancer drugs, thereby facilitating cancer progression [25]. Several studies have shown that microbiota from the gut also translocate to tumour tissue forming the tumour microbiome [24,26–29], suggesting that changes in the gut microbiome could potentially affect the tumour microbiome and the local effects of the latter on tumour biology. Thus, studying the gut (faecal) microbiome is a potentially useful, non-invasive approach to assessing treatment effects on cancer progression. Given the above, we wanted to investigate if HGF/c-MET inhibition ± gemcitabine affected the diversity and composition of the gut microbiome of tumour-bearing mice and how it compared to normal mice.

Thus, we conducted a study involving an immunocompetent mouse model bearing syngeneic orthotopic tumours, where the mice were treated with a combination of HGF/c-MET inhibitors + gemcitabine only after the tumours were sizable and well-established (to simulate the clinical scenario where most patients present at an advanced tumour stage). In addition, given the importance of the gut microbiome that has been implicated in the pathogenesis and immunosuppression of PC, we also investigated whether HGF/c-MET inhibition ± gemcitabine affected the diversity and composition of the gut microbiome of tumour-bearing mice compared to normal (non-tumour bearing) mice.

## 2. Material and methods

### 2.1. Cells

Murine KPC pancreatic cells, which harbor the Kras^LSL−G12D/+^, Trp53^LSL−R172H^, Pdx1-cre mutations, were kindly provided by Professor Paul Timpson of the Invasion and Metastasis Lab at the Garvan Institute in Sydney. These cells were cultured in DMEM supplemented with 10% FBS and 1% antibiotics from Gibco. Mouse PSCs (mPSCs) were isolated from the pancreas of C57BL6 mice using a density gradient isolation method following a previously published protocol [14] and grown in IMDM supplemented with 10% FBS and 1% antibiotics from Gibco. The purity of mPSC isolates was confirmed by morphology, positive staining for glial fibrillary acidic protein (GFAP, a PSC selective marker, Fig. S2). Both KPC and mouse PSC cells were maintained at 37 °C in a humidified atmosphere of 5% CO_2_.

### 2.2. In vivo syngeneic orthotopic animal model

**Fig. 1.**
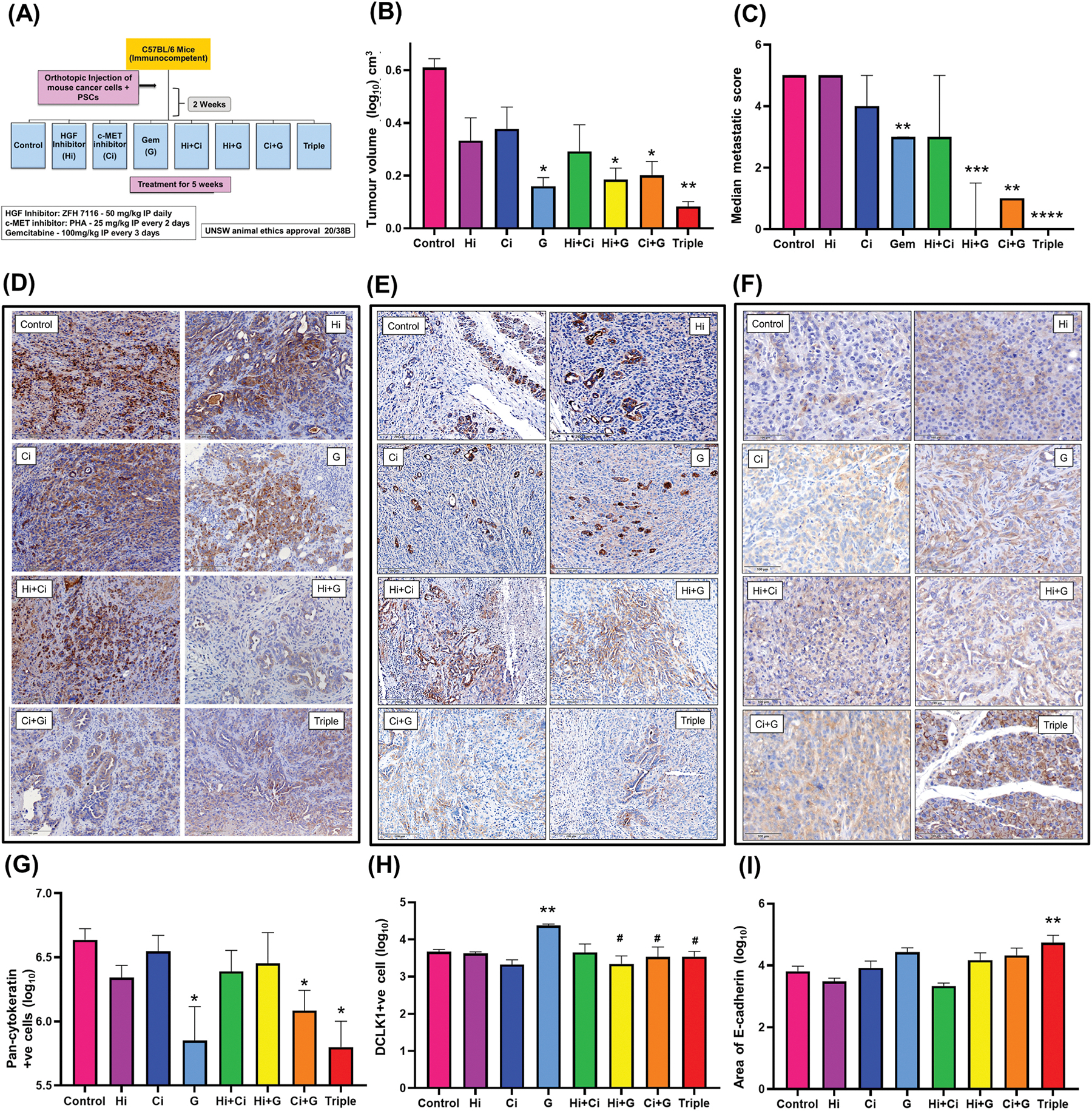
Effects of targeting the HGF/c-MET Pathway ± gemcitabine *in* vivo (A) Experimental design for the syngeneic orthotopic model. (B) Graph showing tumour volume in mice treated with various therapies. Gemcitabine alone and in combination with HGF inhibitor and c-MET inhibitor significantly reduced tumour volume compared to control. (**p < 0.01, *p < 0.05 vs Control, n = 7 to 9 animals/group) (C) Graph showing metastatic score in mice treated with various therapies. Gemcitabine alone and in combination with HGF inhibitor and c-MET inhibitor significantly reduced metastatic score compared to control, with triple therapy virtually eliminating visible metastasis. (****p < 0.0001, ***p < 0.001, **p < 0.01 vs Control, n = 7 to 9 animals/group) (D) Representative images of pan-cytokeratin-stained sections in untreated and treated mice. (E) Representative images of stemness (DCLK1) in control mice and mice treated with single, dual, and triple combinations of HGF inhibitor (Hi), c-MET inhibitor (Ci), and Gemcitabine (G). (F) Representative images of E-cadherin staining in control mice and mice treated with single, dual, and triple combinations of Hi, Ci, and G. (G) Graph showing cancer cell density (pan-cytokeratin) in mice treated with various therapies. Gemcitabine alone and in combination with c-MET inhibitor and triple therapy significantly reduced cancer cell density compared to control. (*p < 0.05 vs Control, n = 7 to 9 animals/group) (H) Graph showing cancer cell stemness (DCLK1) in control mice and mice treated with single, dual, and triple combinations of Hi, Ci, and G. Gemcitabine increased cancer cell stemness, but this effect was significantly reduced in the presence of Hi and/or Ci (*p < 0.05 vs Control, #p < 0.05 vs Gem, n = 7 to 9 animals/group). (I) Graph showing E-cadherin staining in control mice and mice treated with single, dual, and triple combinations of Hi, Ci, and G. E-cadherin was significantly increased only in the triple therapy compared to the control (**p < 0.01 vs Control, n = 7 to 9 animals/group).

The Animal Care and Ethics Committee (ACEC) at The University of New South Wales approved the animal studies under ACEC 20/38B. Female C57BL/6 mice, aged 8–10 weeks, were orthotopically injected with a mixture of KPC and mPSC cells in a 1:1 ratio, consisting of 250 cells each. All mice had detectable tumours by day 10 (as assessed by ultrasound) after cell implantation. Uniformity of tumour size at the time of commencement of treatment, was confirmed by ultrasound measurements of tumour volume using the formula (length × breadth × width)/2. Treatment was initiated between days 10–14 post-implantation. The mice were then treated for a further 5 weeks with either vehicle control, HGF inhibitor (ZFH-7116, Department of Biochemistry and Molecular Biophysics at Washington University in St. Louis, USA), c-MET inhibitor (PHA665752, Selleck Chemicals LLC, TX, USA), gemcitabine (Hospira, Mulgrave, VIC, Australia) as individual agents, or dual and triple combinations as depicted in Fig. 1A. Mice receiving the same treatment were housed at a density of two mice per cage. For gut microbiome studies, faecal samples were collected before and after the treatment (details in Section 2.7.1).

Control: 2.5% DMSO in normal saline (vehicle control for HGF and c-MET inhibitors) administered via intraperitoneal injection daily. Hi: The HGF inhibitor, ZFH-7116, was dissolved in 2.5% DMSO in normal saline at 50 mg/kg and administered via intraperitoneal injection daily. The dose of ZFH-7116 was based on previous studies [30–32]. The details of the compound are presented in supplementary material (Fig. S3 and Table S1).

Ci: The c-MET inhibitor, PHA-665752, was dissolved in 2.5% DMSO in normal saline at 25 mg/kg and administered via intraperitoneal injection every two days. The dosage of PHA-665752 was based on a previous study by Yang et al. [33] on K-ras^LA1^ mice.

Gem: Gemcitabine was diluted in normal saline at 100 mg/kg and administered via intraperitoneal injection every three days. The dosage of gemcitabine was based on a previous study by Hessmann et al. [34] using KPC mice.

Hi + Ci: HGF inhibitor + c-MET inhibitor

Hi + Gem: HGF inhibitor + gemcitabine

Ci + Gem: c-MET inhibitor + gemcitabine

Triple: HGF inhibitor + c-MET inhibitor + gemcitabine

At the end of the experiment, the mice were euthanised, and primary tumours were resected. Samples of tumour, spleen, liver, kidneys, intestine, lung, heart, and thymus were collected and fixed in 10% neutral buffered formalin. Tumour size was measured using digital Vernier calipers, and tumour volume was calculated using the formula (length × breadth × width)/2. The metastatic score was calculated by counting the number of observable metastatic nodules present in both regional locations (such as the spleen, mesentery, and retroperitoneum) as well as distant locations (including the liver, diaphragm, and lungs). A score between 0 (indicating an absence of nodules) and 5 (representing over 20 nodules) was assigned based on the number of identified nodules.

### 2.3. Immunohistochemical analysis

Five micrometre thick serial sections of formalin-fixed and paraffin-embedded pancreas tissue were deparaffinised in xylene and rehydrated in ethanol. Antigen retrieval was performed using citrate buffer pH 6.0 for 20 min, and for immune cell markers using Tris-EDTA buffer pH 9.0. Endogenous peroxidase was blocked using hydrogen peroxide (1%) in methanol for 30 min, followed by blocking with 10% normal goat serum in TBS pH 7.5 for 30 min. Tissue sections were then incubated with mouse reactive primary antibodies against specific markers overnight at 4 °C (Table S2). After washing with TBS pH 7.5 containing 0.05% Tween-20, the sections were incubated with secondary antibodies for 30 min at room temperature and developed using 3,3-diaminobenzidine (DAKO, Agilent Technologies) and counterstained with hematoxylin (DAKO, Agilent Technologies). To quantify the DAB positive area/cells within each image, 20x magnification images of multiple sections covering the entire section were captured using Precipoint M8 microscope/slide scanner and analysed using FIJI ImageJ software [35]. The area/number of cells per section was normalised to the area of the section. For E-cadherin alone, the signal was quantified only in the region of cancer cells (epithelial cells) excluding the stromal regions from the analysis.

### 2.4. In vitro studies

### 2.4.1. Indirect co-cultures

#### 2.4.1.1. Proliferation of KPC cells in response to indirect co-culture.

To measure the effects of HGF inhibition on KPC proliferation, we used the Cell Counting Kit-8 reagent, which produces a formazan when reduced that is directly proportional to the number of living cells. KPC cells (5000 cells/well) were incubated in a 96-well plate (in triplicate) with pre-treated co-cultured conditioned medium (CM) from mPSCs for 18 h at 37 °C. Then, 20 μL of Cell Counting Kit-8 reagent was added to each well, and after 6 h of further incubation, absorbance values were measured at 450 nm using a SpectraMax M2e Microplate Reader (Molecular Devices, Sunnyvale, CA, USA) to assess proliferation (Fig. 3A).

##### 2.4.1.2. Migration of KPC cells in response to indirect co-culture.

To assess the effects of pre-treated co-cultured conditioned media (CM) from mouse pancreatic stellate cells (mPSCs) on the migration of KPC cells, a cell scratch/wound healing assay was performed using the IncuCyte ZOOM^™^ live cell imaging system. KPC cells were grown to confluence in a 96-well plate and uniform scratches were made in all wells using IncuCyte scratch/wound healing assay kit. After washing, 200 μL of pre-treated co-cultured CM was added to triplicate wells. The plate was then placed in the IncuCyte ZOOM^™^ apparatus and images of cell spread was recorded at 0 (baseline) and 24 h. The scratch area at 0 h and 24 h was calculated using Fiji ImageJ software by measuring the area of the remaining vacant space in pixels. The difference between the areas of paired images at 0 h and 24 h was expressed as % migration (Fig. 3A).

##### 2.4.1.3. Apoptosis of KPC cells in response to indirect co-culture.

To induce apoptosis, KPC cells were cultured overnight in serum-free DMEM at a density of 15,000 cells per well in a 96-well plate (in triplicate). Then, the cells were treated with co-cultured CM from mPSCs (as presented in Fig. 3A) in triplicate for 24 h. Apoptosis was measured using Annexin–V-FITC staining as per the Multi-parameter Apoptosis Kit instructions (Cayman Chemicals, Ann Arbor, MI, USA). To count the total number of cancer cells, DAPI staining of cell nuclei was used, while the number of apoptotic cells was determined by counting the fluorescent cells under an Olympus IX71 inverted fluorescent microscope. The results were presented as the percentage of Annexin V positive [apoptotic] cells to DAPI positive (total) cells.

##### 2.4.1.4. Cell signalling pathways mediating KPC cell functions.

KPC cells at 80% confluence were treated for 8 min with i) culture medium, ii) co-cultured conditioned medium (CM) from mouse pancreatic stellate cells (mPSCs), iii) co-cultured CM from mPSCs + HGF inhibitor, iv) co-cultured CM from mPSCs + c-MET inhibitor, and v) co-cultured CM from mPSCs + HGF and c-MET inhibitors (Fig. 3A). Gemcitabine was excluded from the signalling studies because of its direct cytotoxic effects on cancer cells. After treatment, the cells were lysed, and their protein content was estimated using a BCA protein assay kit. The activation of MAPK and PI3K pathways in KPC cell lysates was measured using ELISA kits following the manufacturer’s instructions (ERK1/2 pT202/Y204 + Total) (AB176660) and AKT 1/2/3 pS473 + Total (AB126433) ELISA kits, Abcam Pty Ltd, Melbourne, VIC, Australia).

##### 2.4.1.5. Cytokine secretion of KPC cells in response to indirect co-culture.

To understand the mechanisms by which HGF/c-MET inhibitors may increase CD8^+^ T-cell infiltration as observed in the *in vivo* model, secretion of selected cytokines (TGF-β, TNF-α, IL-1β, IL-6, IL-10) by KPC cancer cells in response to pre-treated mPSC conditioned media were measured. [Note: We excluded Gemcitabine pre-treatment of medium collected from mPSCs due to its cytotoxic effects on cancer cells that could confound cytokine production results]. KPC cells were incubated with pre-treated mPSC secretions for 8 h at 37 °C to minimise the influence of apoptosis on cytokine production. Cytokine concentration in the cell culture supernatant was analysed using ELISA following manufacturer’s instructions (Fig. S7A). The cytokine concentration was normalised to the protein concentration of the supernatants. The limit of detection of the ELISA for each cytokine is presented in Table S3.

### 2.5. Direct co-cultures

#### 2.5.1. Tumour spheroid formation and disintegration

To examine the impact of HGF/c-MET inhibition with or without gemcitabine on spheroid formation at an early stage, KPC and mPSC cells were cultured on a 3D scaffold at a density of 5000 cells each per well in a 96 well plate. The culture medium (DMEM/F-12) included the following treatments: HGF inhibitor (Hi) - ZFH-7116 at 50 μg/mL, c-MET inhibitor (Ci) - PHA-665752 at 25 μg/mL, and gemcitabine (Gem) at 100 μg/mL, as single, dual, and triple combinations. The cells were then incubated at 37 °C for 72 h (Fig. S7B). To study the effect of HGF/c-MET inhibition with or without gemcitabine on pre-formed/established spheroids, KPC and mPSC cells were seeded at a density of 5000 cells each/well in a 3D scaffold 96-well plate with DMEM/F-12 containing 10% FBS and allowed to form spheroids for 72 h. After this, the medium was replaced with DMEM/F-12 containing 0.1% FBS and treatments [HGF inhibitor (Hi) – ZFH-7116 50 μg/mL; c-MET inhibitor (Ci) – PHA-665752 – 25 μg/mL; gemcitabine (Gem) – 100 μg/mL] and incubated for another 72 h at 37 °C. Spheroid formation was evaluated by counting the number and measuring the size of spheroids, and spheroid disintegration was assessed by plotting volume distribution curves for each treatment. Bright field images were captured at the end of early and late interventions using an Olympus ix71 inverted microscope.

### 2.6. Gut microbiome

#### 2.6.1. Faecal sampling and DNA extraction

Mice (n = 8/group) receiving the same treatments were housed in pairs to prevent cross-contamination of the microbiome through coprophagy between mice from different treatment groups. After 35 days, pooled faecal samples were collected from each pair (n = 4/group) and served as untreated tumour and treatment groups. Faecal samples collected before orthotopic tumour induction served as normal controls. The samples were collected aseptically into sterile cryovials and stored at −80 °C until processing. QiAmp Power Faecal Pro DNA Kit (Cat No. 51804, Qiagen, Maryland, MD, USA) was used to extract bacterial DNA from weighed faecal samples following the manufacturer’s instructions.

#### 2.6.2. 16S rRNA gene sequencing and analysis

The bacterial DNA samples were sequenced by Ramaciotti Centre for Genomics, UNSW, Sydney to analyse for the 16S rRNA gene. The raw sequencing data were processed using the operational taxonomical unit (OUT) reporter v1.1.0-beta (15301bc) pipeline, which is based on Mothur, an open-source software (v1.39.5) [36]. Details of the analysis are presented in supplementary methods S1.1.

#### 2.6.3. Statistical analysis

Data from immunohistochemistry and in vitro studies were presented as means and standard error of means. Logarithmic (log10) transformation was applied to achieve the assumptions of normality and homogeneity of variance. Student’s t-test or one-way ANOVA followed by Tukey’s HSD *posthoc* test were used for pair-wise comparisons of treatment means. GraphPad Prism v9.0 software was used for all analyses, and p < 0.05 was considered significant.

## 3. Results

### 3.1. In vivo studies in immunocompetent mice

For the orthotopic model, two weeks post-surgery, mice were randomised to treatment groups receiving HGF inhibitor (ZFH-7116), c-MET inhibitor (PHA-665752), and gemcitabine (a standard chemotherapeutic agent) as single, dual, or triple combinations thereof. The tumour volumes in all mice at the commencement of treatment were 0.036 ± 0.004 cm3 (mean ± SEM) (Fig. S4).

#### 3.1.1. HGF/c-MET inhibition ± gemcitabine decreases tumour volume, stemness, epithelial mesenchymal transition and eliminates metastasis

Gemcitabine alone and in dual and triple combinations with HGF inhibitor and c-MET inhibitor significantly decreased tumour volume and metastasis compared to vehicle-treated animals (Fig. 1B and C,S5). Of most interest was the finding that triple therapy resulted in the greatest decrease in tumour volume and virtually eliminated metastasis (8 out of 9 mice had no metastasis, with only one mouse showing a single metastatic nodule on the liver). Morphometric analysis of tumour sections immunostained for pan-cytokeratin was used to calculate the density of cancer cells in each treatment group (expressed as the number of pan-cytokeratin positive cells/100 mm^2^). Triple therapy significantly reduced pan-cytokeratin expression compared to vehicle-treated tumours, as did gemcitabine alone or in dual combination with the c-MET inhibitor (Fig. 1D and G). Cancer cell stemness as assessed by DCLK1 staining was significantly increased by gemcitabine alone compared to vehicle-treated controls. Notably, this gemcitabine-induced increase in stemness was significantly negated in the presence of HGF inhibitor and c-MET inhibitor. (Fig. 1E and H). Epithelial-mesenchymal transition in the tumour sections was assessed by E-cadherin, an epithelial marker, and vimentin, a mesenchymal marker. Triple therapy significantly increased E-cadherin compared to Control (Fig. 1F and I), while vimentin was unaffected by all treatments (Figs. S6E and G).

#### 3.1.2. Effect of HGF/c-MET inhibition ± gemcitabine on immune cell infiltration

**Fig. 2.**
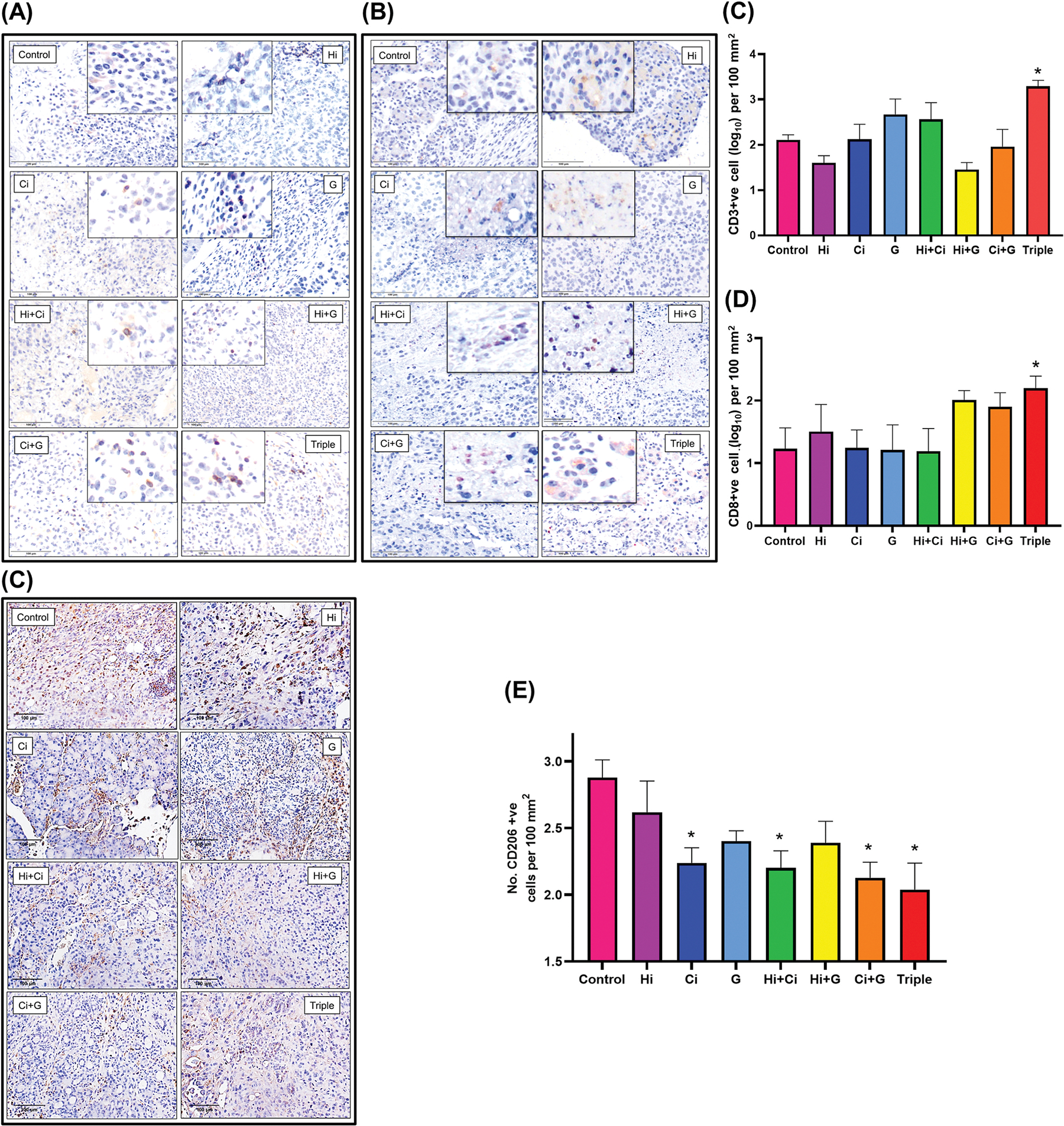
HGF/c-MET pathway inhibition combined with gemcitabine enhances T-cell infiltration in orthotopic mouse model (A) Representative images of CD3^+^ staining showing total T-cell infiltration in tumours from the different treatment groups. (B) Representative images of CD8^+^ staining showing cytotoxic T-cell infiltration in tumours from the different treatment groups. (C) Representative images of CD206+ staining showing M2 type macrophage infiltration in tumours from the different treatment groups. (D) Quantitative graph showing that triple combination therapy significantly increases total T-cell infiltration compared to the control group (*p < 0.05, n = 7 to 9 animals/group). (E) Quantitative graph showing that cytotoxic T-cell infiltration is significantly increased only in the triple therapy group compared to the control group (*p < 0.05, n = 7 to 9 animals/group). (F) Quantitative graph showing that c-MET inhibitor as single, dual and triple combination therapy significantly decreased M2 type macrophage infiltration compared to the control group (*p < 0.05, n = 7 to 9 animals/group).

Only the triple therapy mice showed a significant increase in total T-cell infiltration, as assessed by immunostaining for CD3 (Fig. 2A and C). While there were some differences in helper T-cell infiltration among the treatment groups (as assessed by immunostaining for CD4), these differences were not statistically significant (Figs. S6F and H). A trend towards an increase in cytotoxic T-cell infiltration (as assessed by immunostaining for CD8) was observed in the Hi + G and Ci + G groups, but only the triple therapy group showed a statistically significant increase compared to vehicle-treated (Control) animals (Fig. 2B and D). M2 type macrophages were significantly decreased in all the groups treated with c-MET inhibitor as single, dual and triple combinations compared to vehicle treated animals (Fig. 2B and D). Natural killer cells and MDSCs were not detectable in the tumour sections (data not shown).

#### 3.1.3. HGF/c-MET inhibition ± gemcitabine has no effect on pancreatic stellate cell activation and collagen deposition

We assessed the effect of treatments on the activation of pancreatic stellate cells by performing α-smooth muscle actin staining and picrosirius red staining for collagen deposition (Figs. S6A–D). PSC activation and collagen deposition were unaffected by all treatments when compared to vehicle control.

### 3.2. In vitro studies

#### 3.2.1. Indirect co-culture studies

##### 3.2.1.1. HGF/c-MET inhibition ± gemcitabine decreases cancer cell proliferation and migration and increases apoptosis.

**Fig. 3.**
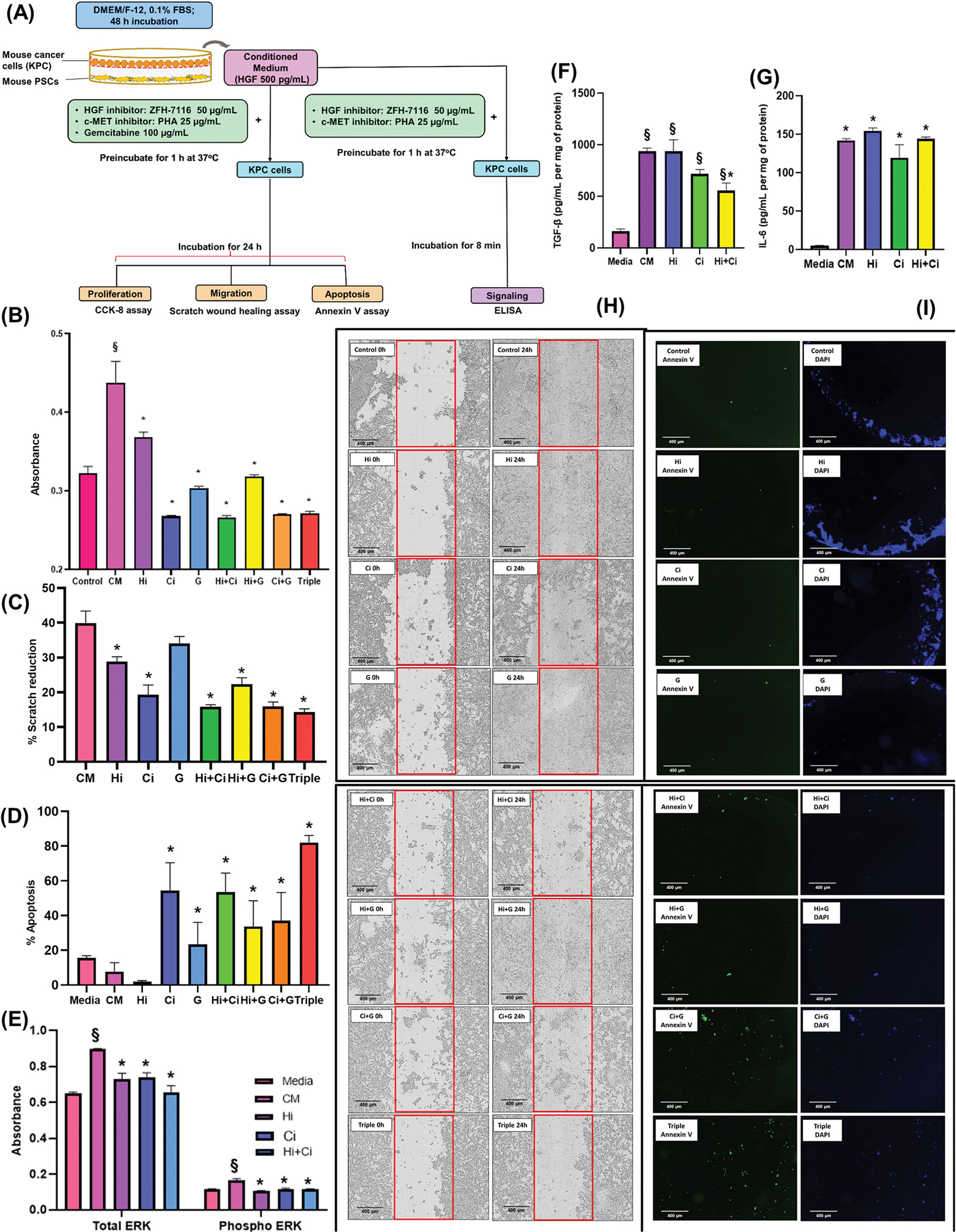
Effect of HGF/c-MET inhibition and gemcitabine on indirect 2D co-culture studies (A) Scheme for in vitro 2D indirect co-culture studies (B) Graph showing the effects of HGF/c-MET inhibition and gemcitabine on KPC cell proliferation in vitro. KPC cells treated with conditioned media (CM) from mPSCs exhibited significantly increased proliferation compared to the cells cultured with untreated medium Control (§p < 0.05 vs medium Control). All treatments significantly decreased mPSC-induced KPC proliferation (*p < 0.05 vs untreated medium Control; n = 3 different mPSC preparations). (C) Graph showing KPC cell migration in response to inhibition of HGF/c-MET and gemcitabine. KPC cells were treated with co-culture conditioned media (CM) from mPSC, which significantly induced KPC migration compared to the Control (§p < 0.05 vs medium Control). All treatments significantly inhibited mPSC-induced KPC migration gemcitabine alone (*p < 0.05 vs conditioned media Control; n = 3 different mPSC preparations). (D) Graph showing the percentage of Annexin V positive KPC cells in response to inhibition of HGF/c-MET and gemcitabine. KPC cells were serum-starved and incubated with mPSC secretions, pre-treated with inhibitors or combinations, and apoptosis was measured. While mPSC secretions significantly inhibited KPC apoptosis compared to Control (§p < 0.05 vs medium Control), c-MET inhibitor as single, dual, or triple combinations significantly increased apoptosis compared to other treatments (*p < 0.05 vs conditioned media Control; n = 3 different mPSC preparations). (E) Graph showing the activation of ERK1/2 in KPC cells in response to inhibition of HGF/c-MET. The graph indicates that mPSC secretions significantly increased total and phosphorylated ERK1/2 (§p < 0.05 vs medium Control) which was significantly inhibited by HGF inhibition, c-MET inhibition, and dual HGF and c-MET inhibition (*p < 0.05 vs conditioned media control, n = 3 secretions from different mPSC preparations). (F) Graph showing TGF-β production by KPC cells in response to inhibition of HGF/c-MET. TGF-β production was significantly increased by mPSC CM compared to medium Control (§p < 0.05 vs medium Control). Treatment with HGF inhibitor or c-MET inhibitor alone did not affect TGF-β production, but the Hi + Ci combination significantly decreased TGF-β production (*p < 0.05 vs conditioned media control; n = 3 different mPSC preparations). (G) Graph showing IL-6 production by KPC cells in response to co-culture with pre-treated mPSC CM. IL-6 production was significantly increased by mPSC CM compared to medium Control (*p < 0.05 vs conditioned media control). Treatment with HGF inhibitor and c-MET inhibitor alone or in combination did not affect IL-6 production by cancer cells (n = 3 different mPSC preparations). (H) Representative images of migration (as assessed by a wound assay) in vitro of KPC cells co-cultured with pre-treated mPSC CM. (I) Representative images of KPC cells undergoing apoptosis (as assessed by Annexin V staining) in response to pre-treated mPSC CM.

To induce maximal proliferation and migration of KPC cells, an HGF concentration of 500 pg/mL in co-culture CM from mPSCs was found in our pilot study to be optimal (data not shown). KPC cells were then cultured in untreated and pre-treated conditioned medium from mPSCs. All treatments (HGF inhibitor, c-MET inhibitor, and gemcitabine as single, dual, and triple combinations) significantly inhibited mPSC-induced KPC proliferation (Fig. 3B). Migration of KPC cells induced by exposure to co-culture CM from mPSC was prevented by all treatments except gemcitabine alone, with the highest reduction observed in KPC cells exposed to triple treatment (Fig. 3C and H). To induce apoptosis, KPC cells were subjected to overnight serum starvation - this was modestly inhibited in the presence of untreated mPSC secretions. There was no effect on cancer cell apoptosis with individual HGF inhibitor and gemcitabine treatments, but apoptosis was significantly increased in the presence of c-MET inhibition as a single agent or in dual combination with HGF inhibitor or gemcitabine. The highest apoptotic effect was observed in the triple combination (Fig. 3D and I).

#### 3.2.2. The effects of HGF/c-MET inhibition on cancer cell functions is mediated via MAPK signalling pathway

To investigate whether ERK and AKT pathways mediate the effects of HGF and c-MET inhibition, KPC cells were exposed to mPSC secretions or secretions pre-treated with ZFH-7116 (HGF inhibitor), PHA-665752 (c-MET inhibitor), or HGF inhibitor + c-MET inhibitor. After 8 min, ELISA assays showed an increase in phosphorylated ERK and total ERK in cancer cells exposed to mPSC secretions. However, both HGF inhibitor and c-MET inhibitor significantly reduced total and phosphorylated ERK levels (Fig. 3E), while phosphorylation of AKT was not affected by any treatment (data not shown).

#### 3.2.3. HGF/c-MET inhibition decreases TGF-β but not IL-6 production by cancer cells exposed to mPSC secretions

KPC cells were stimulated with mPSC secretions for 8 h to induce cytokine production. The presence of mPSC secretions significantly increased TGF-β and IL-6 production by cancer cells. Neither HGF inhibitor nor c-MET inhibitor alone had any effect on TGF-β or IL-6 production by cancer cells. However, inhibition of both HGF and c-MET significantly decreased TGF-β production by cancer cells but did not affect IL-6 production (Fig. 3F and G). TNF-α, IL-1β, and IL-10 were below the limit of detection in all samples, hence the treatment effects could not be assessed.

### 3.3. Direct co-culture studies

KPC cells were directly co-cultured with mPSC to study the effect of HGF/c-MET inhibition ± gemcitabine on the 3D spheroid formation and pre-formed spheroid disintegration.

#### 3.3.1. HGF/c-MET inhibition ± gemcitabine inhibits 3D spheroid formation and increases preformed spheroid disintegration

**Fig. 4.**
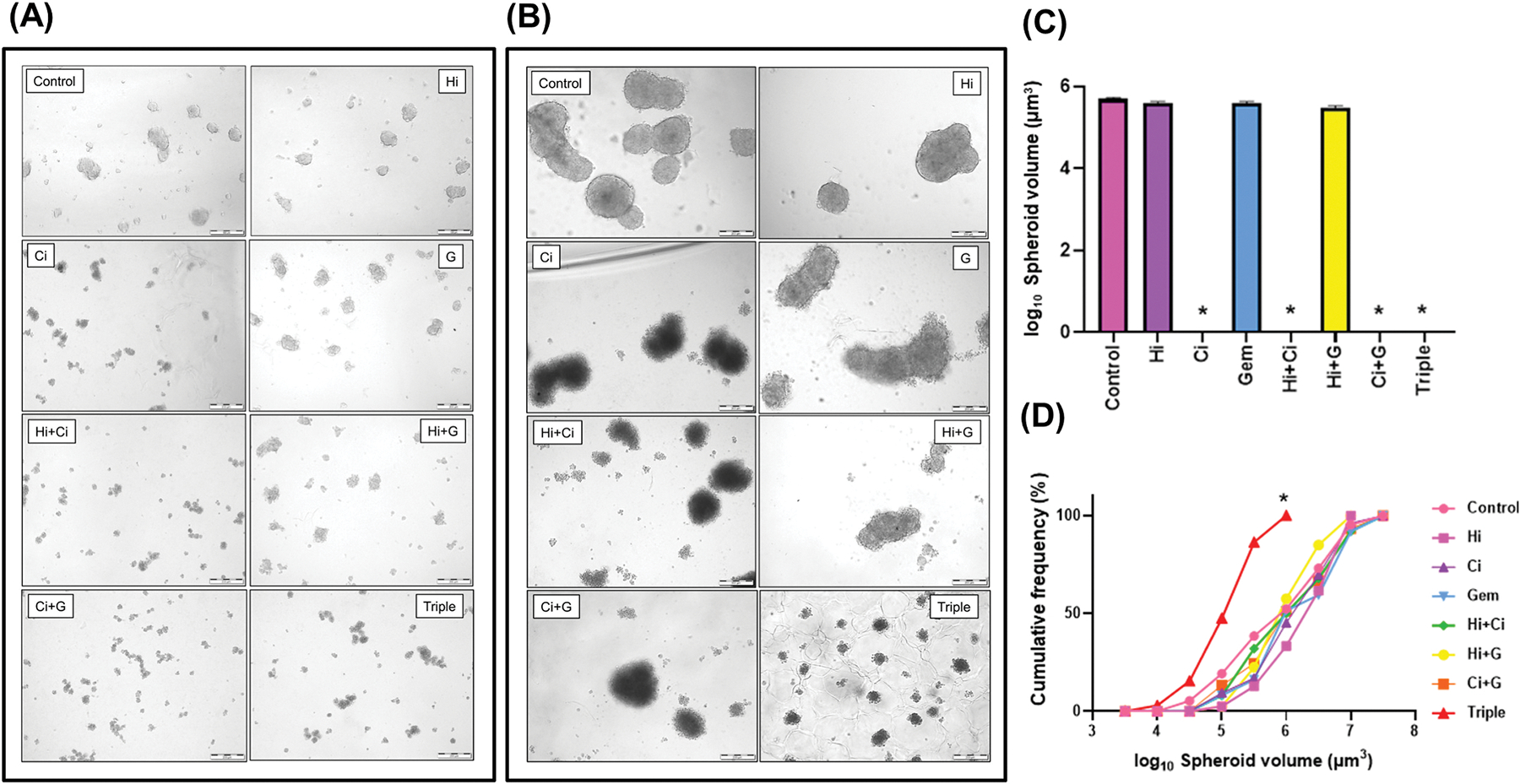
Effect of HGF/c-MET inhibition and gemcitabine on direct 3D co-culture studies (A) Representative images of 3D spheroid formation in response to early treatment with HGF/c-MET inhibitors and gemcitabine (B) Representative images of pre-formed 3D spheroid disintegration in response to late treatment with HGF/c-MET inhibitors and gemcitabine (C) Quantitative graph showing 3D spheroid formation in response to early treatment with HGF/c-MET inhibition and gemcitabine. The co-culture of KPC cells and mPSC with c-MET inhibitor alone or in combination with HGF inhibitor and gemcitabine inhibited spheroid formation compared to Control, while HGF inhibitor and gemcitabine individually and their dual combination did not affect the number and size of the spheroids formed (*p < 0.05 vs Control; n = 3 different mPSC preparations). (D) Quantitative graph showing pre-formed 3D spheroid disintegration in response to late treatment with HGF/c-MET inhibitors and gemcitabine. The co-culture of pre-formed spheroids with HGF inhibitor, c-MET inhibitor, and gemcitabine as single agents or in dual combination did not affect spheroid disintegration. However, only triple therapy successfully disintegrated pre-formed spheroids, leading to a shift to the left in the volume distribution curve compared to Control (*p < 0.05 vs Control; n = 3 different mPSC preparations).

The effect of different treatments on the formation and disintegration of spheroids was evaluated in two separate studies. In the early intervention study, it was found that HGF inhibitor and gemcitabine alone did not affect the size and volume of the spheroids formed. However, c-MET inhibitor alone or in combination with HGF inhibitor and gemcitabine completely inhibited the formation of spheroids (Fig. 4A and C). In the late intervention study, HGF inhibitor and gemcitabine alone or in dual combination had no effect on the disintegration of pre-formed spheroids, while c-MET inhibitor alone or in combination with HGF inhibitor and gemcitabine merely caused minor dissociation of spheroids. It was only triple therapy that appeared to result in disintegration of pre-formed spheroids, demonstrated by a significant shift to the left in the volume distribution curve (Fig. 4B and D).

### 3.4. Gut microbiome

The diversity and composition of gut microbiota were analysed using 16S rRNA gene sequencing of fecal samples collected from mice at day 0 (prior to implantation of KPC + mPSCs) and at the end of the 5-week treatment period. This was done to examine the effects of tumour development (untreated tumour-bearing mice vs normal mice) and the effects of HGF/c-MET inhibition ± gemcitabine (treatments vs untreated tumour-bearing mice) on gut microbiota. A total of 4,897,436 reads were obtained after data processing and quality checking, with an average of 132,300 reads per sample. These sequences were clustered into operational taxonomic units (OTUs), resulting in 64,931 OTUs with an average of 1762 OTUs per sample.

#### 3.4.1. Gut microbial composition

**Fig. 5.**
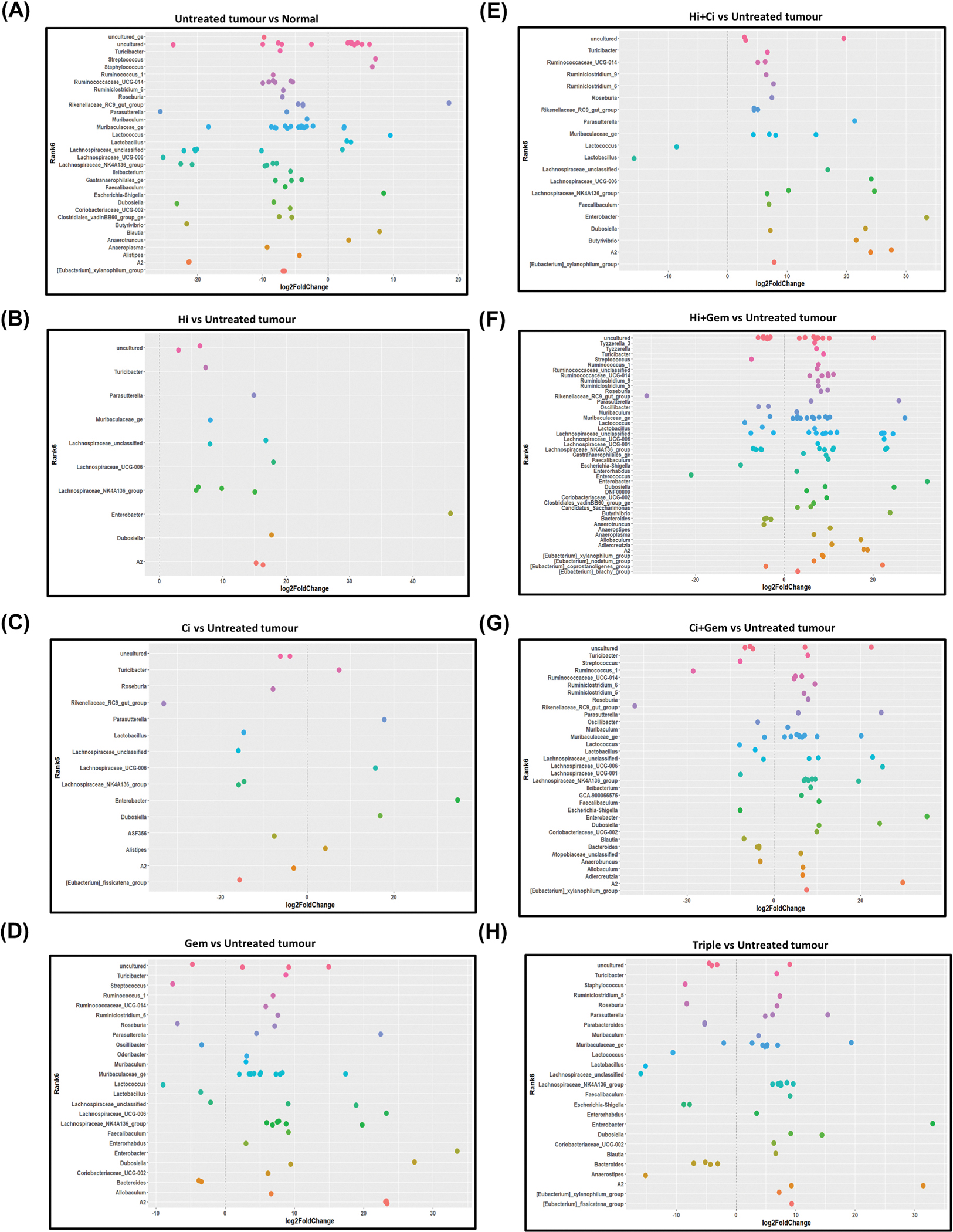
Differential taxa expression at genus level in various treatments of tumour-bearing mice (A) Untreated tumour control mice compared to normal mice. Untreated control groups showed an increase in 17 typical genera, one pathogenic genus, and a decrease in 11 typical gut microbiota genera. Different colours represent different genera. (Wald Chi-Squared Test; p < 0.01; n = 4/group). (B) Hi-treated mice vs untreated tumour-bearing mice. Hi-treated mice showed a significant increase in 10 typical gut microbiota genera. Different colours represent different genera. (Wald Chi-Squared Test; p < 0.01; n = 4/group). (C) Ci-treated mice vs untreated tumour-bearing mice. Ci increased six typical genera and decreased nine typical gut microbiota genera. Different colours represent different genera. (Wald Chi-Squared Test; p < 0.01). (D) Gem-treated mice vs untreated tumour control mice. Gem increased 20 typical gut microbiota genera and decreased six typical gut microbiota genera and one pathogenic genus compared to the untreated tumour group. Different colours represent different genera. (Wald Chi-Squared Test; p < 0.01). (E) Hi + Ci treated mice vs untreated tumour control mice. Hi + Ci increased 18 typical gut microbiota genera and decreased two typical gut microbiota genera compared to the untreated tumour group. Different colours represent different genera. (Wald Chi-Squared Test; p < 0.01). (F) Hi + Gem treated mice vs untreated tumour control mice. Hi + Gem treatment increased 37 typical gut microbiota genera and decreased 14 normal gut microbiota genera and three pathogenic genera compared to the untreated tumour group. Different colours represent different genera. (Wald Chi-Squared Test; p < 0.01). (G) Ci + Gem treated mice vs untreated tumour control mice. Ci + Gem treatment increased 23 typical genera and decreased 14 typical and two pathogenic genera compared to the untreated tumour group. Different colours represent different genera. (Wald Chi-Squared Test; p < 0.01). (H) Triple treated mice vs untreated tumour-bearing mice. Triple therapy increased 17 typical genera and decreased 13 normal and two pathogenic genera compared to the untreated tumour-bearing mice. Different colours represent different genera. (Wald Chi-Squared Test; p < 0.01).

Gut microbiota composition in normal, tumour-bearing mice and treatment groups at phylum and class level is presented in the supplementary material (S2 Results). At the genus level, analysis of the fecal microbiota of untreated tumour-bearing mice revealed an increase in pathogenic bacteria such as Escherichia-Shigella, Streptococcus, and Staphylococcus, as well as a decrease in core gut microbiota including Parasutterella, Ruminococcaceae, Faecalibaculum, Ileibacterium, and Alistipes, compared to normal mice (Fig. 5A). Treatment effects on specific genera, compared to untreated tumour-bearing mice, are presented in supplementary results (S2.1c).

##### 3.4.1.1. Alpha diversity.

**Fig. 6.**
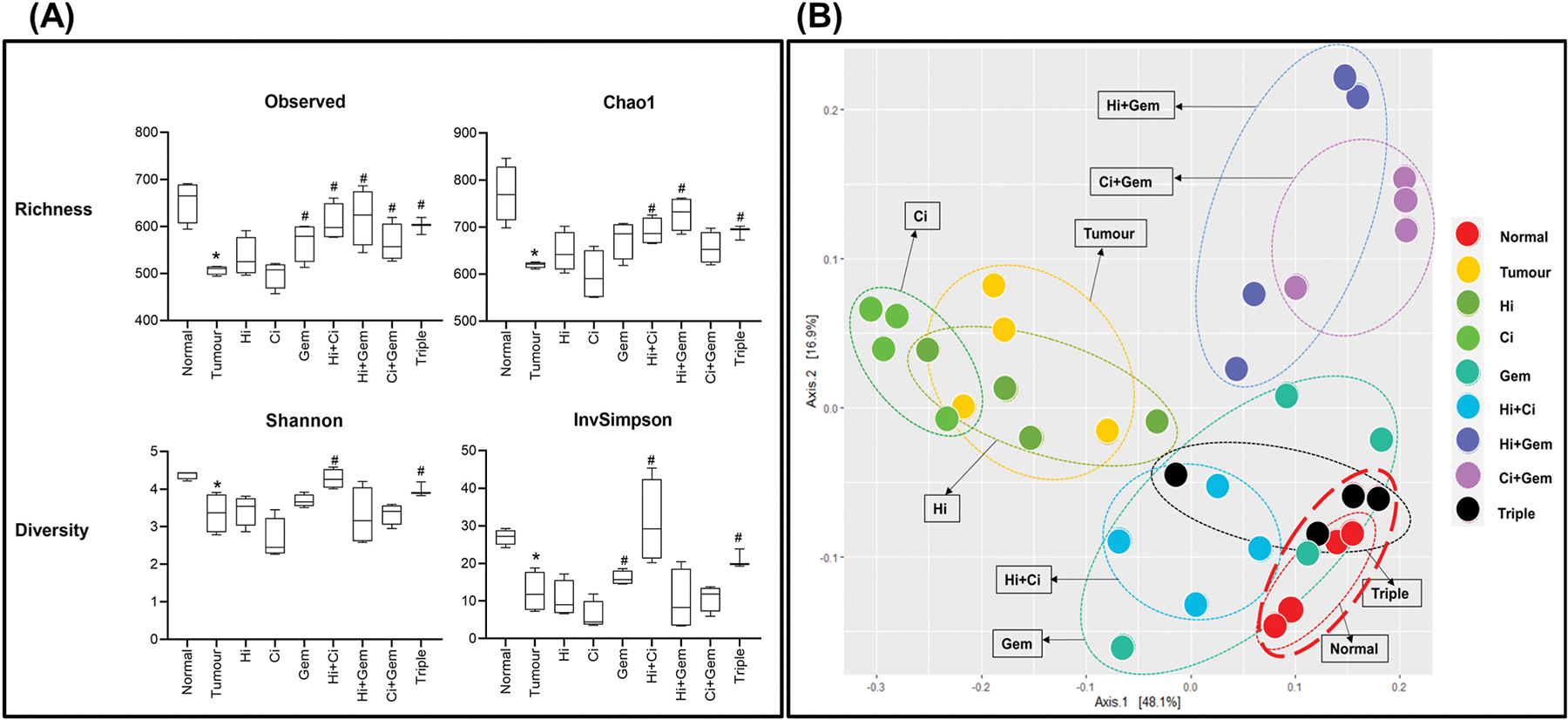
Effect of HGF/c-MET inhibition combined with chemotherapy on gut microbiota composition and diversity in pancreatic cancer (A) Graph showing alpha diversity indices (n = 4/group) in normal mice, untreated tumour-bearing mice, and mice treated with HGF inhibitor, c-MET inhibitor, and gemcitabine alone or in combination. Tumour-bearing mice had significantly decreased community richness and diversity indices compared to normal mice. Treatment with gemcitabine, Hi + Ci, Hi + Gem, Ci + Gem, and triple therapy significantly increased richness indices. Diversity indices were significantly increased only by Hi + Ci and triple therapy (*p < 0.05 vs normal group; #p < 0.05 vs untreated tumour group; n = 4 samples per group). (B) Weighted UniFrac principal co-ordinate analysis plot (n = 4/group) used to compare the dissimilarity/similarity of microbiota composition of normal mice, untreated tumour-bearing mice, and mice treated with HGF inhibitor, c-MET inhibitor, and gemcitabine alone or in combination. The untreated tumour group had a significantly different microbiota composition compared to normal mice. All treatments (except Hi alone) significantly changed the microbial composition compared to tumour-bearing mice. Triple therapy was most effective in normalising gut microbiota, as three out of four mice treated with triple therapy clustered close to normal mice. (Pairwise ADONIS with 10,000 permutations, p < 0.05).

Tumour-bearing mice showed a significant decrease in community richness (Observed and Chao1) and diversity indices (Shannon and Inverse Simpson) compared to normal mice, as determined by alpha diversity analysis. Treatment with gemcitabine, Hi + Ci, Hi + Gem, Ci + Gem, and triple therapy significantly increased the richness (Observed and Chao1) when compared to untreated tumour-bearing mice. However, only Hi + Ci and triple therapy significantly increased diversity indices (Shannon and Inverse Simpson) (Fig. 6A).

##### 3.4.1.2. Beta diversity.

We conducted a principal coordinates analysis (PCoA) using weighted UniFrac distances to determine the intra-group differences in the gut microbial community after the treatment protocol. The axes of the plot accounted for 65% of the treatment variation (48.1% on axis 1 and 16.9% on axis 2). The composition of the microbiota in tumour-bearing mice was significantly different from that of normal mice (ADONIS with 10,000 permutations, p = 0.001).

Pair-wise ADONIS test was also conducted, which revealed that all treatments, except for Hi alone, significantly altered the microbial composition compared to tumour-bearing mice. Notably, we observed that three of the four mice who underwent triple therapy clustered closely with the normal mice in plot. This finding suggests that triple therapy was the most effective treatment in restoring the gut microbiome to a normal state (Fig. 6B).

## 4. Discussion

In this study, we used an immunocompetent syngeneic orthotopic model of pancreatic cancer, wherein tumours were produced by the implantation of mouse cancer (KPC cells) + mouse PSCs into the pancreas. Given that PSC-cancer cell interactions drive PC progression and that the HGF/c-MET pathway is one of the mediators of this interaction, we used a therapeutic approach involving HGF and/or c-MET inhibition with and without a chemotherapeutic agent (gemcitabine). We found that the combination of all three interventions (i.e., HGF inhibitor + c-MET inhibitor + gemcitabine, referred to as triple therapy) resulted in the greatest reduction in tumour volume and, importantly, in virtual elimination of metastasis, the driver of mortality in PC. Notably, triple therapy significantly increased total and cytotoxic T-cell infiltration into the tumours and also improved gut microbial diversity while normalising the microbial composition.

Tumour size is influenced by the number of cancer cells and their rate of growth. We found that triple therapy resulted in significant reduction in the expression of cytokeratin, which is a marker that reflects the number of cancer cells in the tumour. Gemcitabine administered alone or in combination with HGF or c-MET inhibitors also reduced tumour volume to some extent. The observed decrease in tumour volume *in vivo* could thus result from i) direct inhibitory effects on cancer cell proliferation and increased apoptosis leading to decreased cancer cell density ii) ability of triple therapy to disintegrate established spheroids as demonstrated in in vitro experiment and ii) increased cytotoxic T-cell infiltration. In the in vitro studies, specifically, we observed that triple therapy decreased cancer cell proliferation and increased apoptosis supporting the *in vivo* findings. We found that HGF inhibition alone did not significantly affect apoptosis of cancer cells, compared to cells treated with mPSC secretions, while c-MET inhibitor alone significantly increased cancer cell apoptosis. This differential effect may be related to the fact that c-MET binds a number of ligands other than HGF, including macrophage-stimulating protein (MSP), laminin, Galectin-3, MSP-alpha, and epithelial growth factor receptor-related protein (EGFR), many of which have been shown to inhibit cancer cell apoptosis [37–40]. It is possible therefore that c-MET inhibition prevents these anti-apoptotic effects of additional ligands, while HGF inhibition alone is unable to increase apoptosis because of the putative presence of the other ligands in mPSC secretions. Furthermore, 3D studies have demonstrated that only treatment with triple therapy resulted in the effective disintegration of pre-formed spheroids. This finding is consistent with our *in vivo* observations of reduced tumour volume and elimination of metastasis. In a previous study, it was reported that c-MET inhibitor (PHA-665752), was effective in reducing the growth of PDAC spheroids. However, the study only used cancer cells and did not include the supporting stromal cells [41].

Although gemcitabine demonstrated efficacy in reducing tumour volume, our studies indicate that it also induces stemness in pancreatic tumours. This suggests that gemcitabine may select a subset of cancer cells with stem cell-like properties, potentially contributing to the recurrence of pancreatic cancer. Our findings corroborate previous studies, which showed that PC cells surviving gemcitabine treatment expressed increased markers of stem cell differentiation leading to increased migration and resistance to chemotherapy [42–44]. Furthermore, a similar increase in stemness has also been reported in PC cell lines exposed to FOLFIRNOX [45]. Interestingly, combining gemcitabine with the HGF inhibitor or c-MET inhibitor or as part of the triple combination negated the induction of stemness by gemcitabine. This reversal may be explained by the fact that the emergence of stemness and EMT in cancer cells is reported to be mediated via the c-MET receptor [46] and inhibiting its stimulation directly (via a c-MET receptor inhibitor) or indirectly via inhibition of its ligand HGF, prevents the effects of c-MET on stemness, thereby neutralising the stemness-inducing effect of gemcitabine.

With respect to metastatic dissemination, gemcitabine alone or in combination with either an HGF inhibitor or a c-MET inhibitor, reduced the spread of cancer cells to distant sites compared to the tumour-bearing mice that did not receive any treatment. However, our most notable finding, which holds significant implications for the clinical outcome of the disease, was that the combination of HGF and c-MET inhibition with gemcitabine (triple therapy) exerted a synergistic effect resulting in virtual elimination of visible metastasis to distant organs. Metastasis is a complex process which involves several mechanisms, including EMT, invasion, migration, evasion of immune response, angiogenesis, extravasation, colonisation and proliferation. In this study, we found that triple therapy i) significantly decreased EMT in the *in vivo* tumours and ii) reduced migration of cancer cells (in the presence of PSC conditioned medium). These mechanisms offer a potential explanation for the observed elimination of metastasis in the triple treated group. Previously c-MET receptor expression was shown to increase the resistance of cancer cells to gemcitabine [46–48]. c-MET has been shown to activate the antioxidant transcription factor Nuclear factor-erythroid factor 2-related factor 2 (NRF2) – haemoxygenase1 (HO-1) pathway, thereby countering chemotherapy-induced reactive oxygen species (ROS) generation and apoptosis of cancer cells [49]. Interestingly, gemcitabine treatment has also been shown to activate NRF2, thereby promoting chemoresistance [50,51]. Hence, combining HGF and c-MET receptor inhibitors with gemcitabine could also sensitise cancer cells to the direct killing effects of gemcitabine, leading to a synergistic decrease in tumour volume and eliminating metastasis. Further investigation of the relationship between Nrf2 activation and the response to our triple regimen could provide valuable insights into the underlying mechanisms of the therapy. Our finding of synergism *in vivo* support those reported previously by in vitro studies. Crizotinib (c-MET antibody) was found to synergistically increase the in vitro antiproliferative and apoptotic effect of gemcitabine on Capan-1-gemcitabine-resistant cells [52]. Similarly, tivantinib also showed synergistic anti-tumour activity in combination with gemcitabine on cancer cells with high c-MET expression [53].

Our in vitro studies demonstrated a significant reduction in the activation of mitogen-activated protein kinase (MAPK) pathway (which regulates cancer cell proliferation, migration, and survival) by the HGF inhibitor, c-MET inhibitor, and their combination. Furthermore, previous research reported similar outcomes in the human pancreatic cancer cell line ASPC-1, where cancer-associated human pancreatic stellate cells induced the phosphorylation of extracellular signal-regulated kinase (ERK), but not protein kinase B (AKT) [54].

The immune cell profile of pancreatic cancer (PC) is characterised by a decrease in the infiltration of cytotoxic CD8+ve T-cells and an increase in the regulatory component of helper T-cells (CD4+ve Treg) [55]. In our study, we found that only the combination of HGF inhibitor, c-MET inhibitor, and gemcitabine significantly increased the infiltration of total CD3+ve T-cells and cytotoxic CD8+ve T-cells in pancreatic tumours *in vivo*. Our in vitro experiments revealed that the dual combination of HGF inhibitor and c-MET inhibitor significantly reduced the secretion of TGF-β by cancer cells. This decrease could be due to (i) a decrease in cancer cell numbers secondary to induction of apoptosis by HGF/c-MET inhibition (Fig. 3D). (ii) inhibition of MAPK signalling as discussed below (Fig. 3E). Since TGF-β is a mediator for immunosuppression, a decrease in its expression may mediate the observed increase in cytotoxic T-cell infiltration in the triple therapy group, thus providing a possible explanation for the observed increase in cytotoxic T-cell infiltration in that group. Previous research has indicated that c-MET receptors may interact with programmed cell death protein 1 (PD-L1), facilitating immunosuppression [56]. Therefore, inhibiting the c-MET receptor in our study could have led to increased T-cell infiltration. Moreover, gemcitabine is known to increase CD8+ve T-cell infiltration by enhancing antigen presentation and immune checkpoint inhibition while concurrently inhibiting TGF-β [57]. Thus, the triple therapy combination of HGF inhibitor, c-MET inhibitor, and gemcitabine increased the infiltration of total T-cells and cytotoxic T-cells in orthotopic tumours.

In PC, tumour-associated macrophages (subset of M2 macrophages) play a crucial role in promoting immunosuppression, cancer cell stemness, metastasis, and drug resistance [58,59]. In our study, we observed a significant reduction in M2 macrophage infiltration in the tumours of all groups treated with a c-MET inhibitor (administered as a single agent, in combination with HGF inhibitor or c-MET inhibitor, or in a triple combination) compared to the control mice. This finding suggests that inhibiting c-MET receptors could have contributed to the decrease in M2 macrophage infiltration, as the HGF/c-MET signalling pathway is one of the key drivers responsible for the polarization of M1 to M2 macrophages [60,61]. In our experimental model, we observed that the administration of triple therapy did not affect the activation of pancreatic stellate cells (PSCs) or the development of fibrosis. This suggests that neither the inhibition of HGF pathway nor c-MET influenced the function of PSCs. This finding is in line with previous research that has demonstrated the absence of c-MET receptors on PSCs [62].

In this study, we evaluated the impact of HGF/c-MET inhibition + gemcitabine on the gut microbiome, given the growing recognition of its role in cancer biology. We found that untreated tumour-bearing mice showed reduced microbial diversity with an increased presence of pathogenic bacteria and a decreased Bacteroides to Firmicutes ratio. These findings concur with reports of decreased Bacteroides to Firmicutes ratio and increased proteobacteria and gammaproteobacterial in PC patients, suggesting their potential role in disease progression [63–66]. The presence of pathogenic bacteria such as Bacteroides, Escherichia, Shigella, and Staphylococcus in PC patients has also been reported, indicating their potential in the progression of PC [67,68]. We observed that alpha diversity, a measure of intra-sample richness and evenness of bacterial species, was significantly increased by gemcitabine alone or in combination with HGF inhibitor and c-MET inhibitor, and triple therapy. We also found that the microbiome of untreated tumour-bearing mice clustered far away from normal healthy mice based on principal coordinate analysis using weighted UniFrac distances. However, the microbiome of animals receiving triple treatment clustered close to that of normal mice, indicating the normalisation of the gut microbiota in the triple therapy group. In addition, we observed a decrease in the abundance of pathogenic bacteria with gemcitabine alone or in combination with HGF inhibitor or c-MET inhibitor and triple therapy. These findings suggest that HGF/c-MET inhibition + gemcitabine has a positive impact on the gut microbiome, which may contribute to improved clinical outcomes in PC patients.

The findings of this study indicate that targeting the HGF/c-MET signalling pathway in combination with gemcitabine may have a synergistic effect in inhibiting pancreatic cancer growth and promoting anti-tumour immunity. Additionally, the results suggest that this combination therapy can positively impact the gut microbiome by increasing diversity and normalising the microbial composition.

Normalising the gut microbiome holds promising clinical implications in the management of pancreatic cancer. It has been shown that specific bacterial species can metabolise chemotherapeutic agents, thereby affecting drug bioavailability and response [69]. Modulating the gut microbiome composition could potentially enhance the effectiveness of chemotherapy and reduce its adverse effects. Preclinical studies have also demonstrated that modulating the gut microbiome through the administration of probiotics or fecal microbiota transplantation (FMT) can inhibit tumour growth, enhance response to chemotherapy, and improve overall survival [70,71]. Although the specific mechanisms underlying the gut microbiome’s impact on pancreatic cancer are yet to be fully elucidated, research to date suggests that normalising the gut microbiome through targeted interventions is a promising strategy to complement current therapeutic approaches in pancreatic cancer.

### 4.1. Possible mechanisms of action of triple therapy on pancreatic cancer progression via both cancer cells and pancreatic stellate cells

PSCs secrete Pro-HGF which is activated by proteases such as hepatocyte growth factor activator and urokinase plasminogen activator (uPA). This active HGF binds to c-MET receptors on cancer cells and triggers downstream signalling through MAPK, PI3K, and STAT3 pathways. This signalling regulates cancer cell proliferation, migration, and survival. The binding of HGF to c-MET receptors also increases the synthesis of uPA by cancer cells, leading to a feed-forward loop that promotes cancer progression.

In pancreatic cancer, immune suppression is facilitated by two factors: i) TGF-β secreted by cancer cells which prevents the infiltration of cytotoxic T-cells into the tumour, and ii) gut microbial dysbiosis caused by pathogenic organisms which activate inflammation via toll-like receptors, leading to tumour progression and differentiation of tumour-associated macrophages into immune-suppressive M2 phenotype.

**Fig. 7.**
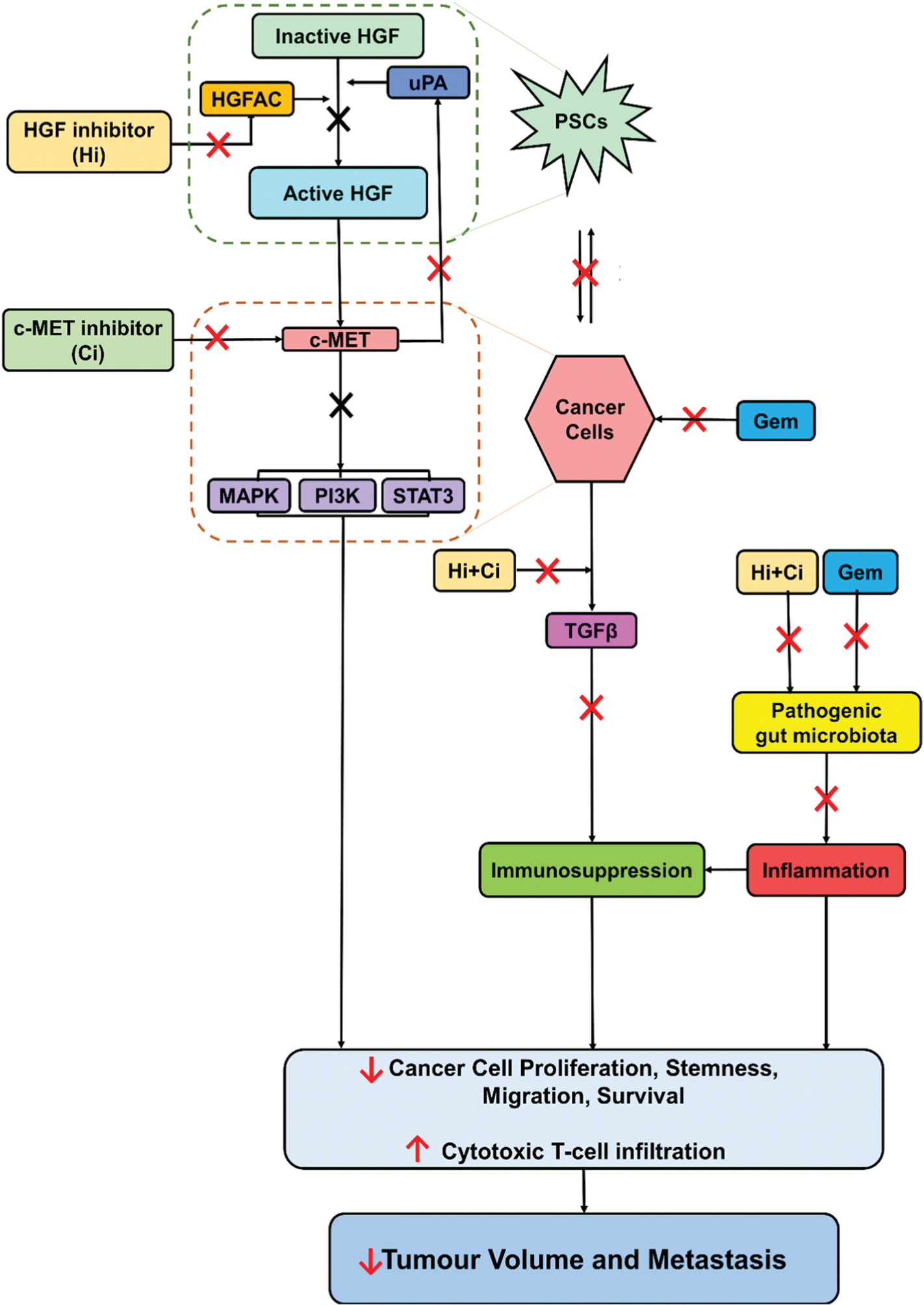
Possible mechanisms of HGF/c-MET inhibition + gemcitabine on pancreatic cancer progression and tumour immunity. 1. HGF inhibitor ZFH-7116 stops the conversion of pro-HGF to active HGF by inhibiting the activator protease HGFAC. 2. c-MET inhibitor PHA-665752 blocks the c-MET receptor, which decreases its ability to bind with its ligand. This results in (i) reduced downstream signalling pathways (MAPK, PI3K, and STAT3), which leads to reduced cancer cell proliferation, migration, and survival; and (ii) blocked uPA synthesis and the feed-forward loop. 3. HGF and c-MET inhibitors prevent bidirectional interactions between cancer cells and pancreatic stellate cells, and they also stop gemcitabine-induced cancer cell stemness. Additionally, these agents (i) inhibit the secretion of TGF-β, which is the primary immunosuppressive cytokine in pancreatic cancer. By inhibiting TGF-β, the agents increase cytotoxic T-cell infiltration into tumours. (ii) Decrease pathogenic bacteria in the gut, which could reduce their facilitatory effects on tumour growth and potentially decrease their translocation to the pancreas. 4. Gemcitabine directly kills cancer cells and can also reduce TGF-β levels in the tumour. It can also decrease pathogenic bacteria in the gut. Overall, the combination of the HGF/c-MET pathway inhibitors and chemotherapy (gemcitabine) significantly reduces local tumour growth and virtually eliminates visible metastasis. HGF = hepatocyte growth factor; HGFA = hepatocyte growth factor activator; uPA = urokinase plasminogen activator; PSCs = pancreatic stellate cells; c-Met = mesenchymal-epithelial transition factor; TGF-β transforming growth factor beta; MAPK = mitogen-activated protein kinase; PI3K = phosphoinositide 3-kinase; STAT3 = signal transducer and activator of transcription 3; Gem = gemcitabine; X = inhibition.

The levels at which the triple therapy targets the above-described pathways, and their effects are depicted in Fig. 7.

## 5. Conclusions

In conclusion, this study provides robust evidence that targeting the HGF/c-MET pathway in combination with chemotherapy reduces cancer progression (by inhibiting tumour growth and metastasis), increases anti-tumour immunity, and improves the diversity of the gut microbiome. The findings of this study have important clinical implications, as they suggest a potential novel therapeutic pathway for pancreatic cancer. Specifically, the availability of HGF and c-MET inhibitors (namely, human HGF neutralising antibody YYB 101 (CellabMED Inc. Seoul, Korea) [72] and the c-MET inhibitor, Capmatinib (INC280) (Novartis) [73] that are currently in clinical trials for other malignancies, indicates that our proposed triple therapy approach (using the above inhibitors + the routinely used chemotherapeutic agent gemcitabine) is an eminently feasible treatment option for clinical testing in pancreatic cancer.

## Supplementary Material

Supplementary material

## Data Availability

All data and materials are published in the manuscript, supplementary materials on the journal website or available on request.

## Appendix A. Supplementary data

Supplementary data to this article can be found online at https://doi.org/10.1016/j.canlet.2023.216286.
