# Supplementary material for: HGF/c-Met pathway inhibition combined with chemotherapy increases cytotoxic T-cell infiltration and inhibits pancreatic tumour growth and metastasis
Source: Cancer Lett. Author manuscript; Available in PMC 2026 Feb 6. (PMC12880582; doi:10.1016/j.canlet.2023.216286)
Supplement: Supplementary material [file NIHMS2134887-supplement-Supplementary_material.docx]

Supplementary Information

**HGF/c-Met Pathway Inhibition Combined with Chemotherapy Increases Cytotoxic T-cell infiltration and Inhibits Pancreatic Tumour Growth and Metastasis**

Alpha Raj Mekapogu, Zhihong Xu, Srinivasa Pothula, Chamini Perera, Tony Pang, S.M.Zahid Hosen, Vishnu Damalanka, James Janetka, David Goldstein, Ron Pirola, Jeremy Wilson, Minoti Apte

Inventory of Supplementary Information

Supplemental Material and Methods

Supplemental Results

Supplemental Figures S1-S8

Supplemental Tables S1-S3

**S1 Material and Methods**

**S1.1 Gut microbiome Analysis**

**S1.1.1 16S rRNA gene sequencing and analysis**

Bacterial DNA samples were sequenced for the 16S rRNA gene and analysed by the Ramaciotti Centre for Genomics, UNSW, Sydney, as per their in-house protocol, as detailed below.

1. **Library preparation**

PCR barcoding mix for bacterial 16S rRNA:

1. 1 U IMMOLASE™ DNA Polymerase (Bioline)
2. 2.5 µL of ImmoBuffer (PCR buffer, Bioline)
3. 2.5 mM MgCl2
4. 0.2 mM dNTPs
5. 0.5 µM of each primer - Barcoded 27F (268) and 519R (269)
6. 1 µL of DNA template
7. **Thermal cycling conditions:** (All PCRs were conducted in 25 µL volumes)
8. Initial denaturation at 95° C for 10 min
9. 35 cycles of denaturation at 94° C for 30 s
10. Annealing at 55° C for 10 s
11. Elongation at 72° C for 45 s
12. Final elongation at 72° C for 10 min

PCR products were normalised and pooled using the SequalPrep™ Normalization Plate Kit (Thermo Fisher Scientific, Australia). The library was purified using the Axygen® AxyPrep™ Mag PCR Clean-Up Kit (Fisher Biotech, Australia) as per the manufacturer’s instructions. The concentration and quality of the pooled library were checked with Qubit® and the library size on an Agilent 2200 TapeStation. Primer dimers were reduced or removed using the Agencourt AMPure XP Bead Clean-up kit. The library pool was sequenced on the MiSeq using a MiSeq Reagent Kit v3 with a 2x300 bp run format, using default run parameters, including adaptor trimming. Custom primers were added to the reagent cartridge for Read1, Index, & Read2.

1. **Community analysis**

Raw sequencing data were processed with the operational taxonomical unit (OTU) reporter v1.1.0-beta (15301bc) pipeline, based on Mothur, an open-source software (v1.39.5). Briefly, the reads were quality-filtered and assigned to their respective samples. Sequences were trimmed according to the MiSeq standard operating procedure, and only sequences between 301 and 532 bp were retained (as the V1 to V3 region length is around 490 bp). Samples with homopolymers longer than eight bp were removed. Chimeric sequences were removed using the chimera V search script included in Mothur (270). The sequences were aligned and classified against the SILVA reference alignment (v132), and lineages not targeted by the primers (*i.e*., Archaea, Chloroplast, Eukaryota, Mitochondria, unknown) were removed. Sequences were grouped into operational taxonomical units (OTUs) based on 97% similarity using the OptiClust algorithm and then subsampled based on the sample with the lowest number of sequences, i.e., 16842 sequences. Sequencing error was assessed using ZymoBIOMICS™ Microbial Community Standard as the control in each sequencing run. Representative sequences for each OTU based on the most abundant sequence were selected with the get. oturep command from Mothur. An unrooted phylogenetic tree was built using FastTree v2.1.11 under the GTR+Gamma model. The resulting tree was utilised to calculate the weighted UniFrac distance matrices. Alpha diversity indices - Observed, Chao1, Shannon, InvSimpson -were calculated using the Vegan package. Beta diversity was assessed by principal coordinates analysis (PCoA) of the weighted UniFrac distances by the Vegan R package. OTU richness plots, diversity plots, and report plots were generated with the phyloseq and ggplot R packages v4.2.1.

1. **Statistical analysis**

For the gut microbiome analysis, ANOVA followed by the Tukey *posthoc* test was used to test the statistical significance of alpha diversity indices. Non-parametric permutation-based multiple ANOVA (ADONIS) was conducted to test the statistical significance of similarity/dissimilarity of treatment groups in weighted UniFrac plots. Pair-wise ADONIS test was used to compare treatment groups with normal and untreated control groups. The DESeq2 R package was used to evaluate differences in the abundance of bacterial genus between treatment groups. All the analyses for gut microbiome were performed in R-studio v4.2.1.

**S 1.2 Genetically engineered transgenic mouse model**

To determine the effects of HGF/c-MET inhibition with and without gemcitabine on the progression of the earliest lesions of pancreatic cancer (Pancreatic Intraepithelial Neoplasms, PanINs), we used a well established transgenic model – the KPC mouse model. The KPC mice were generated by crossing KC mice LSL-KrasG12D/+, Pdx-1Cre mice) (with PC mice (Trp53/+/, Pdx-1Cre), both strains generously provided by Professor Paul Timpson, Garvan Institute, Sydney. At two weeks of age, genotyping of offspring was performed by polymerase chain reaction (PCR) at Garvan Molecular Genetics. All mice were housed at Australian Bioresources, Mossvale, under pathogen-free conditions and ad-libitum access to water and food. At five weeks, KPC mice were transferred to Biological Resources Unit at the Ingham Institute and allowed to acclimatise for one week before commencing the study. All the animal experiments were approved by the Animal Care and Ethical Committee (ACEC) of the University of New South Wales.

**S1.2.1 Experimental design**

Eight-week-old KPC mice were randomised into eight groups and treated with HGF inhibitor, c-MET inhibitor and Gemcitabine, as single dual or triple combinations similar to the regimen used for the orthotopic model described previously (Figure S7C).

**S2 Results**

**S 2.1 Gut microbial composition**

1. **At phylum level**

The faecal microbiota composition of the different treatment groups was analysed at the end of the treatment period of five weeks. Microbiota composition (in normal mice as well as the treatment groups) showed twelve main phyla, of which Firmicutes (58.95 ± 2.30%, overall average), Bacteroides (31.27 ± 1.95%, overall average) and Proteobacteria (8.41 ± 2.23%, overall average) were the most abundant **(Figure S1A)**. Compared to normal mice, the untreated tumour group showed a significant increase in Firmicutes and Proteobacteria and a significant decrease in Bacteroidetes and Bacteroides: Firmicutes ratio **(Figure S1B)**. A reduction in Bacteriodetes and an increase in Firmicutes and Proteobacteria is a characteristic feature of many gastrointestinal diseases and cancers and represents decreased gut microbial diversity.

Compared to the untreated tumour group, treatment effects are as follows: HGF inhibitor (Hi) alone showed no significant effect on all phyla. c-MET inhibitor (Ci) alone significantly increased proteobacteria without significantly affecting other phyla. Gemcitabine (Gem) significantly increased Bacteroides and Bacteroides: Firmicutes ratio (thus reversing the effect seen in the untreated tumour-bearing mice) with no significant effect on Firmicutes and Proteobacteria. Hi+Ci significantly increased Bacteroides, Proteobacteria, and Bacteroides: Firmicutes ratio with no significant effect on Firmicutes. Hi+Gem and Ci+Gem significantly decreased Proteobacteria with no significant effect on Bacteroides, Firmicutes, and Bacteroides: Firmicutes ratio. Triple therapy significantly increased Bacteroides, Proteobacteria, and Bacteroides: Firmicutes ratio with no significant effect on Firmicutes **(Figure S1B)**.

#### At class level

Sixteen classes were found, of which Bacteroidia (31.27 ± 1.95%, on overall average), Clostridia (23.89 ± 1.81%, on overall average), Bacilli (19.68 ± 2.43%, on overall average), Erysipelotrichia (15.39 ± 2.96%, on overall average), and Gammaproteobacteria (GPB) (8.13 ± 2.25%, on overall average) were predominant **(Figure S1C)**. Compared to normal mice, Tumor-bearing mice showed a significant increase in Bacilli and GPB and a significant decrease in Bacteroidia and Erysipelotrichia **(Figure S1D)**.

Compared to the untreated tumour group, treatment effects are as follows: Hi alone showed no significant effect on all classes. Ci alone significantly increased GPB. Gemcitabine significantly decreased Bacilli, Bacteroidia, and Erysipelotrichia with no significant effect on GPB. Hi+Ci and Hi+Gem significantly decreased Bacilli and GPB with no significant effect on Bacteroides and Erysipelotrichia. Ci+Gem significantly decreased Bacilli and GPB and significantly increased Erysipelotrichia. Triple therapy significantly decreased Bacilli and GPB and significantly increased Bacteroidia and Erysipelotrichia **(Figure S1D).**

1. **At genus level**

Compared to untreated tumour-bearing mice, treatment effects on specific genera were as follows: Gemcitabine alone decreased Streptococcus (log2fold change -7.59 to -7.24); Hi+Gem decreased Escherichia-Shigella (log2fold change 8.54 to -9.84) and Streptococcus (log2fold change 7.24 to -7.73); Ci+Gem decreased Escherichia-Shigella (log2fold change 8.54 to -45.91) and Streptococcus (log2fold change 7.24 to -45.91); Triple therapy decreased Escherichia-Shigella (log2fold change 8.54 to -8.55) and Staphylococcus (log2fold change 6.79 to -8.55) **(Figure 5B to 5H)**. However, all treatments increased the abundance of an opportunist pathobiont, Enterobacter.

| **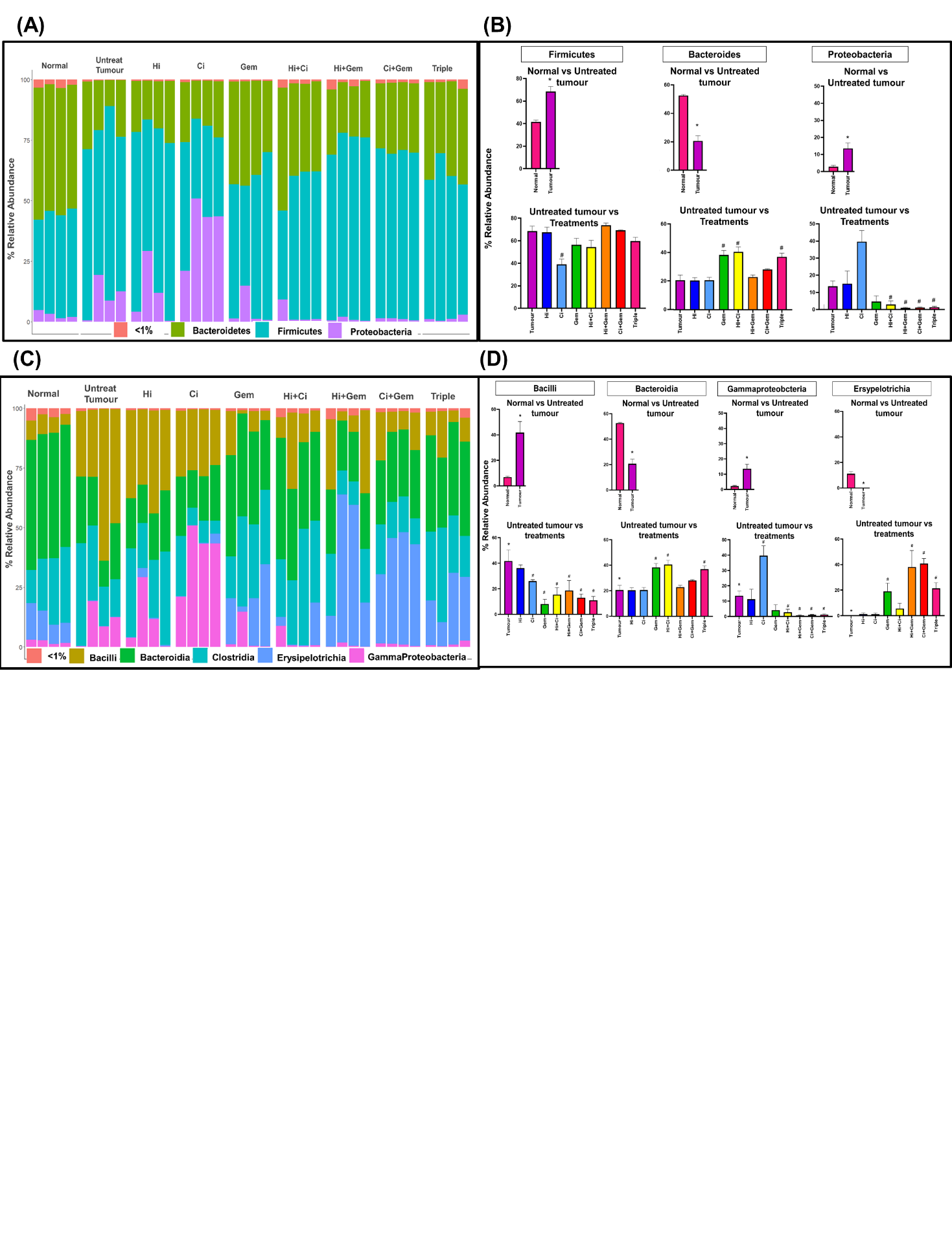** |
| --- |
| **Figure S1 Effect of HGF/c-MET inhibition ± gemcitabine on gut microbiota composition**  (A) Stacked bar graph showing the relative abundance of microbiota at the phylum level in normal mice, untreated tumour-bearing mice, and mice treated with HGF inhibitor, cMET inhibitor, and gemcitabine as single, dual, or triple combinations (n=4/group).  (B) Quantitative graph of the relative abundance of microbiota at the phylum level in normal mice, untreated tumour-bearing mice, and mice treated with HGF inhibitor, cMET inhibitor, and gemcitabine as single, dual, or triple combinations (n=4/group). Compared to normal mice, the untreated tumour group showed a significant increase in Firmicutes and Proteobacteria and a significant decrease in Bacteroidetes. Treatment with Gem, Hi+Ci, and Triple therapy significantly increased Bacteroidetes, while Ci alone, Hi+Ci, Hi+Gem, Ci+Gem, and Triple therapy significantly decreased Proteobacteria. Firmicutes was not significantly affected by any treatment. Hi alone did not show significant effects on any phylum. (*p<0.05 vs normal group; #p<0.05 vs untreated tumour group).  (C) Stacked bar graph showing the relative abundance at class level in normal mice, untreated tumour-bearing mice, and mice treated with HGF inhibitor, cMET inhibitor, and gemcitabine as single, dual, or triple combinations. (n=4/group)  (D) Quantitative graph of the relative abundance of bacteria at class level in normal mice, untreated tumour-bearing mice, and mice treated with HGF inhibitor, cMET inhibitor, and gemcitabine as single, dual, or triple combinations. Compared to normal mice, the untreated tumour group showed an increase in Bacilli and Gammaproteobacteria and a decrease in Bacteroidia and Erysipelotrichia. Lower panel: Bacteroidia was significantly increased by Gem, Ci+Gem, and Triple therapy; Bacilli was significantly decreased by Gem, Hi+Ci, Hi+Gem, Ci+Gem, and Triple therapy; Gammaproteobacteria was significantly decreased by Hi+Ci, Hi+Gem, Ci+Gem, and Triple therapy; Erysipelotrichia was significantly increased by Gem, Ci+Gem and Triple therapy. Hi alone showed no significant effect on all classes and Ci significantly increased Gammaproteobacteria. (*p<0.05 vs normal group; #p<0.05 vs untreated tumour group; n= 4 samples per group).  **S 2.2 Effect of HGF/c-MET inhibition ± gemcitabine on precursor lesion density in KPC mice**  To test whether HGF/c-MET inhibition combined with gemcitabine was sufficient to suppress pancreatic intraepithelial neoplasm formation, pancreatic sections from KPC mice were stained with mucin 5AC (MUC5AC), which is the most abundantly overexpressed mucin during early pancreatic intraepithelial neoplasia. Muc5AC positive PanINs were counted to compute PanIN density in the pancreas **(Figure S2.3 A and B).**  At the end of the treatment period, the pancreas from untreated KPC mice showed multifocal PanIN lesions. However, following treatment with triple therapy, the density of PanIN lesions was significantly decreased compared to the Control. There was also a significant decrease in pan-cytokeratin-stained area in both dual treatments with HGF inhibitor + gemcitabine and triple therapy **(Figure S2.4A and 2.4B)**.  **S 2.3 Effect of HGF/c-MET inhibition ± gemcitabine on T-cell infiltration in KPC mice**  As assessed by CD3 staining, total T-cell infiltration was significantly increased only in triple therapy compared to vehicle-treated (Control) animals. Helper T-cell infiltration was significantly decreased by gemcitabine in dual combination with HGF inhibitor or c-MET inhibitor and triple combination compared to vehicle-treated (Control) animals. HGF-inhibitor, c-MET inhibitor as single and dual combination showed no significant difference compared to Control. Cytotoxic T-cell infiltration was significantly increased by the dual combination of c-MET inhibitor and gemcitabine and triple therapy compared to vehicle-treated (Control) animals. HGF-inhibitor, c-MET inhibitor, gemcitabine as single and dual combinations of HGF inhibitor and c-MET inhibitor, and HGF inhibitor and gemcitabine showed no significant difference compared to Control **(Figure S8 A to H)**. |

**S 3 Supplementary Figures:**

| 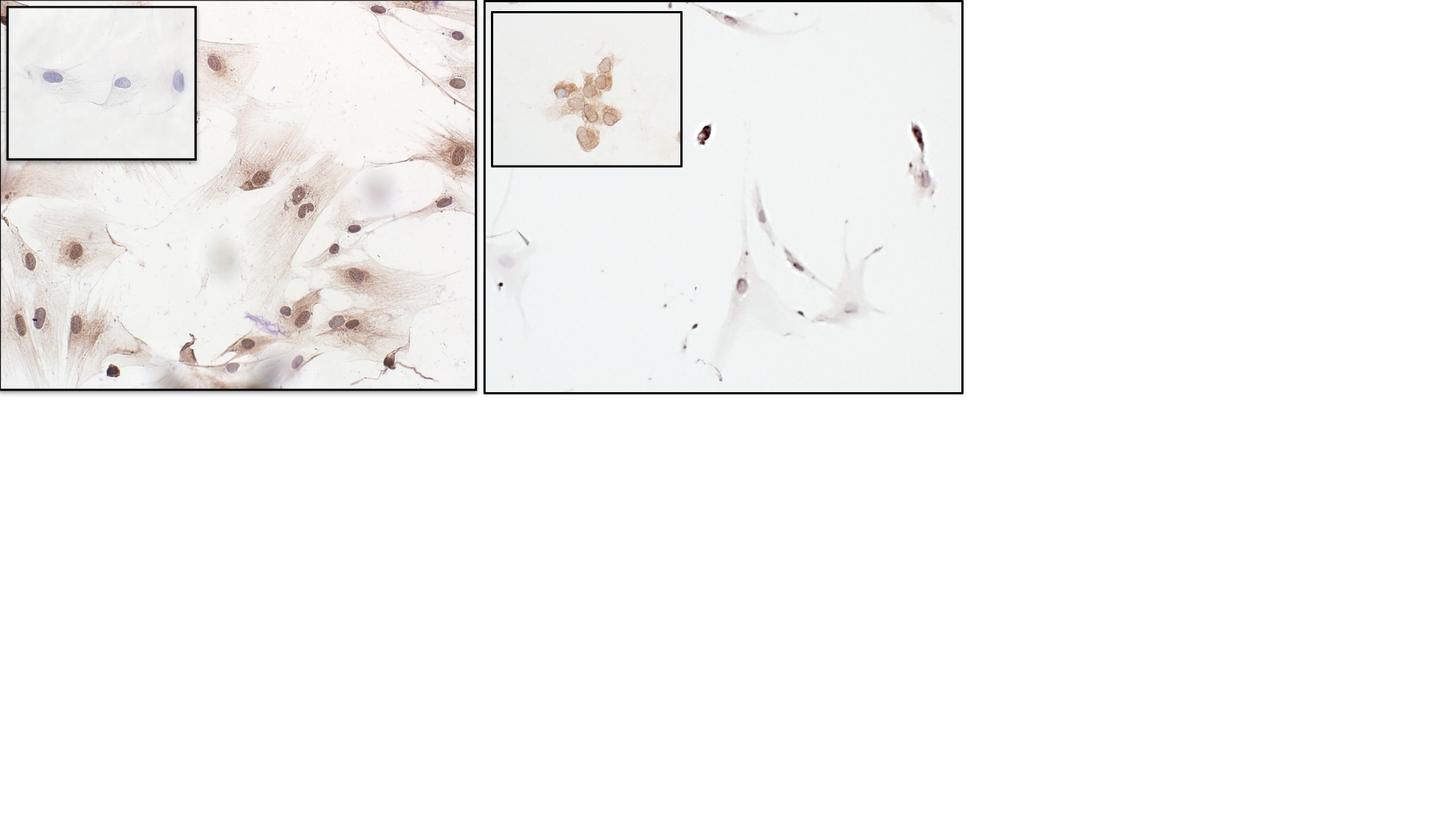 |
| --- |
| **Figure S2** showing the immunostaining results of mPSCs for GFAP (PSC marker). The inset shows a negative control stained by isotype IgG showing no brown staining, and the panel shows mPSCs, which show a positive signal (brown staining) for the PSC-specific marker GFAP. 400x magnification. |

| 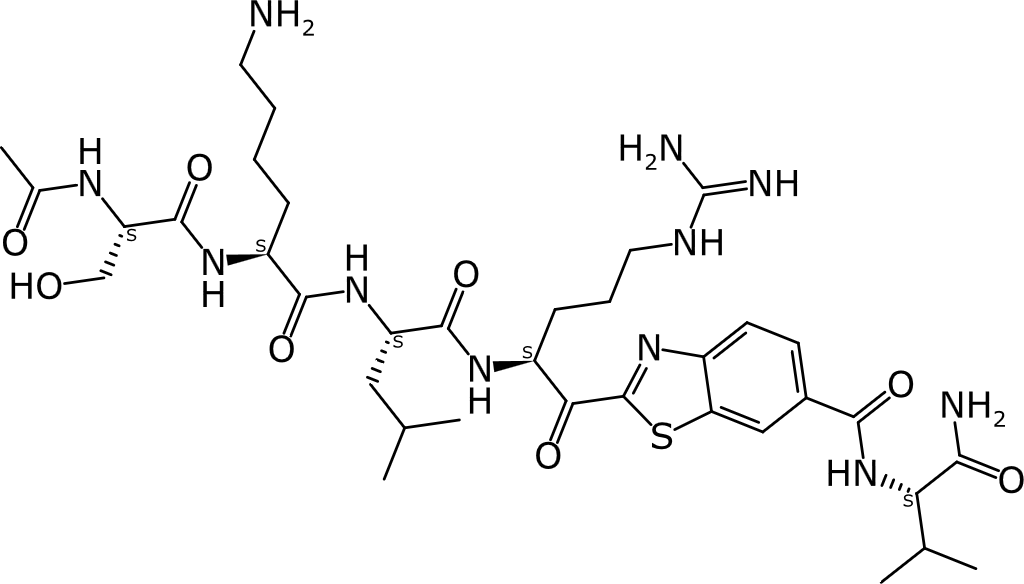 |
| --- |
| **Figure S3 Structure of ZFH-7116 a triplex inhibitor of the proteases (HGFA, Matriptase, Hepsin) involved in the conversion of inactive pro-HGF to active HGF.**  IUPAC name: N-[(S)-1-carbamoyl-2-methylpropyl]-2-[(S)-2-[(S)-2-{(S)-1-[(S)-2-acetylamino-3-hydroxypropionylamino]-5-aminopentylcarbonylamino}-4-methylvalerylamino]-5-guanidinovaleryl]-1,3-benzothiazole-6-carboxamide |
|  |

| **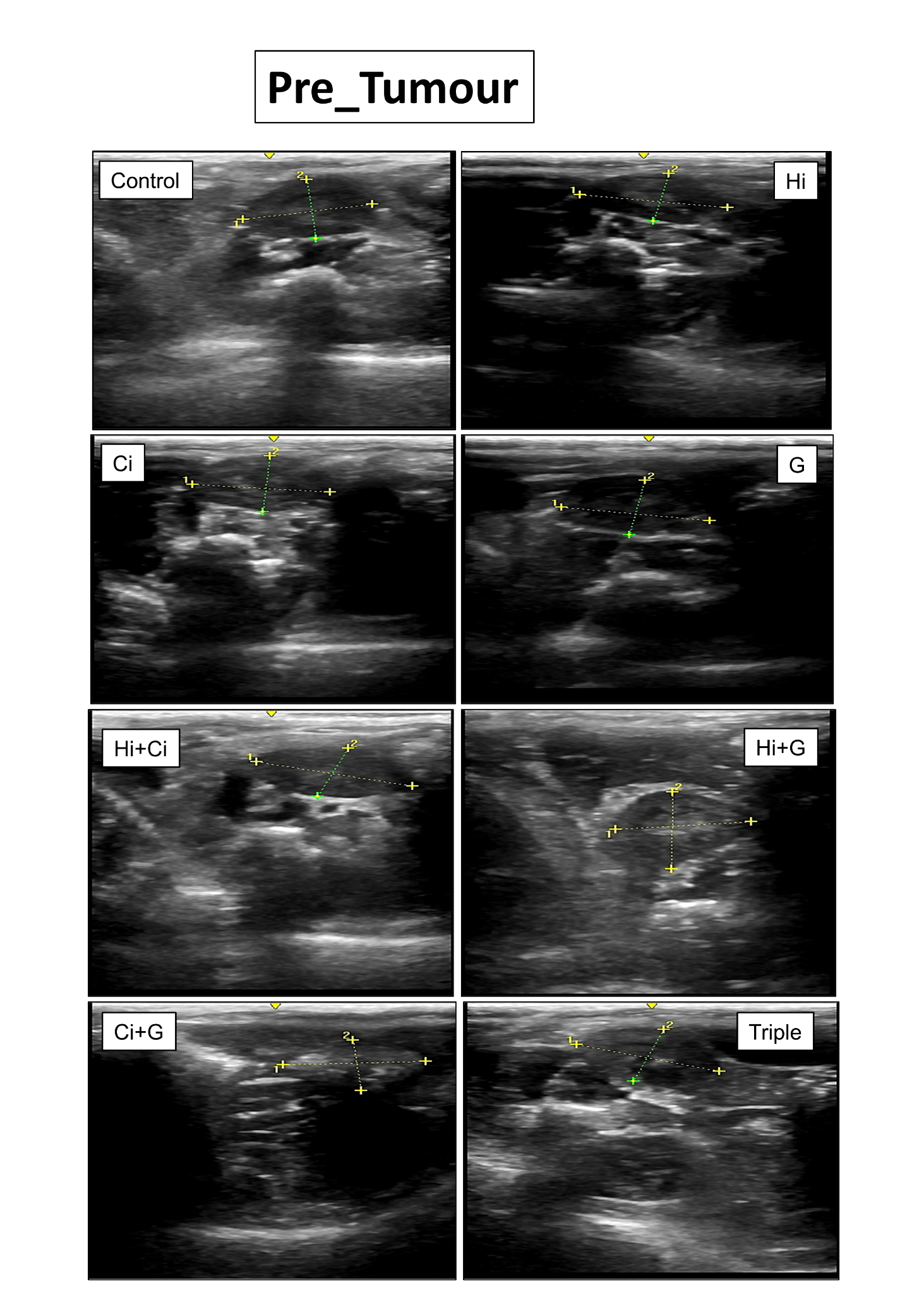** |
| --- |
| **Figure S4 Representative images of pre-treatment tumour volume between 10 to 14 days post-implantation of KPC + mPSC cells into the pancreas of C57BL6 mice. The lines indicate the longest diameters (d1 and d2) for computing tumour size.** |

| **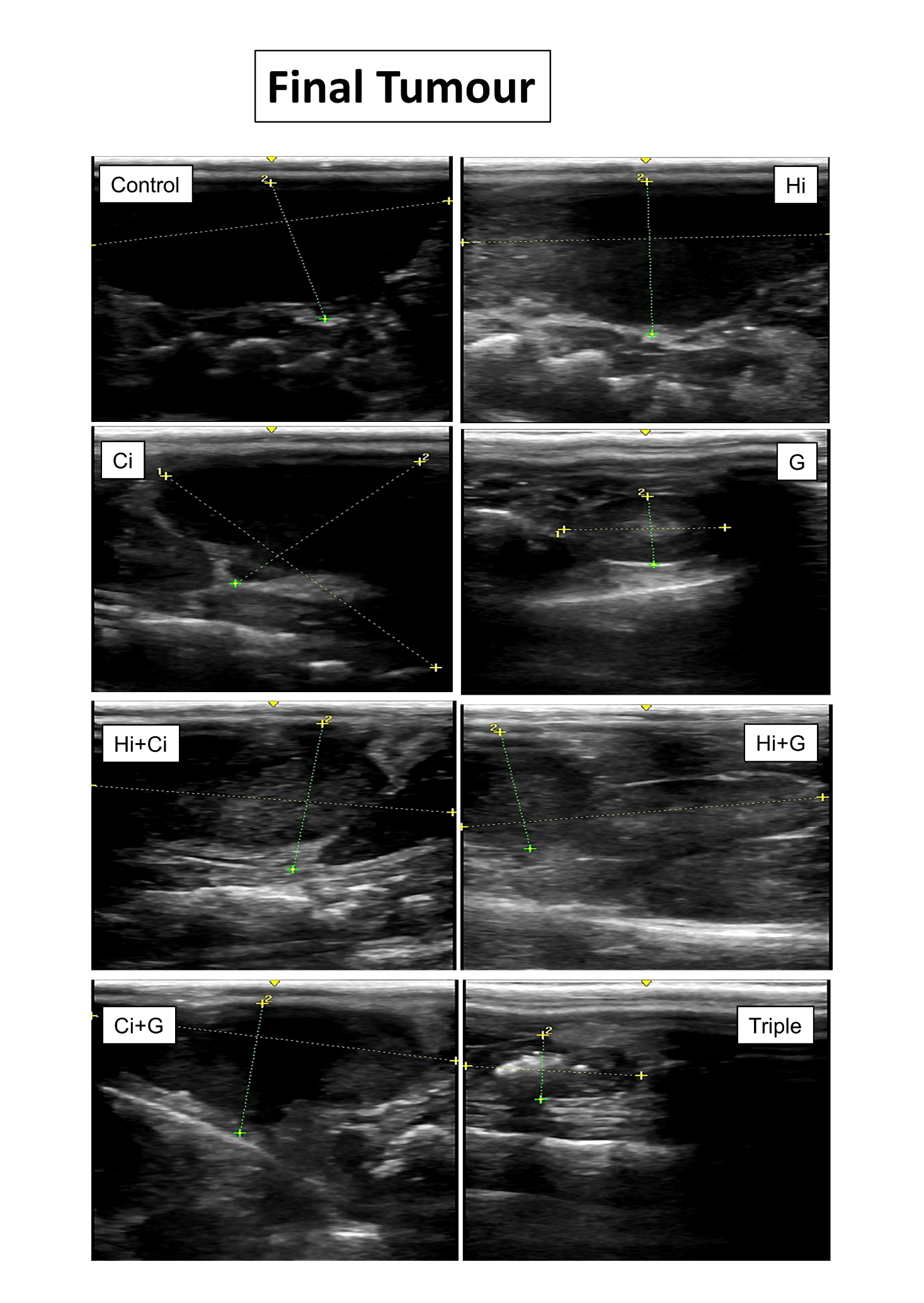** |
| --- |
| **Figure S5 Representative ultrasound images of tumour volume before euthanasia in different treatment groups. The lines indicate the longest diameters (d1 and d2) for computing tumour size.** |

| 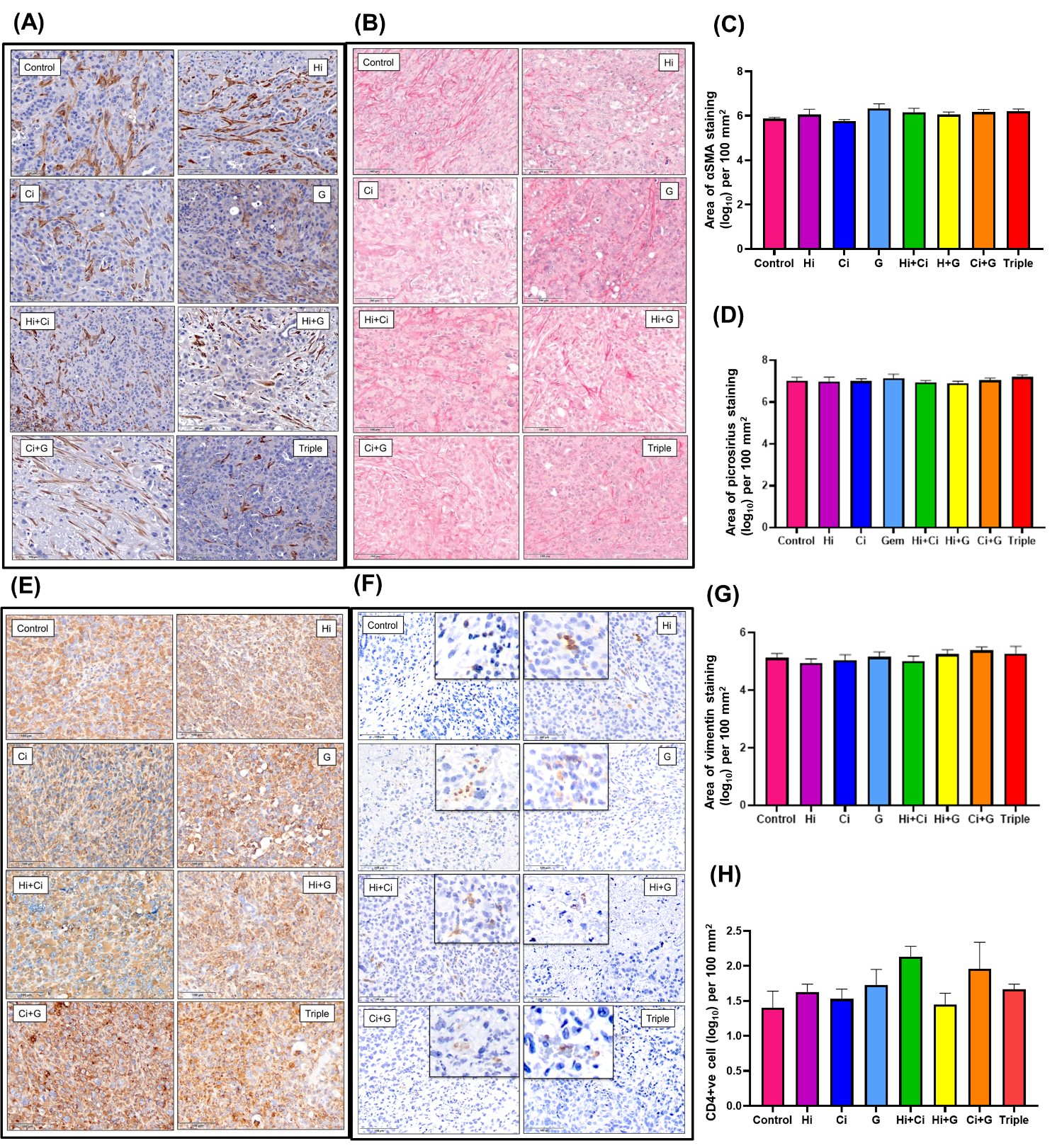 |
| --- |
| **Figure S6 Effects of HGF inhibitor, c-MET inhibitor, and Gemcitabine on α-smooth muscle actin and collagen staining in mice**  (A) The figure shows representative images of α-smooth muscle actin staining in mice that were either untreated (control) or treated with HGF inhibitor (Hi), c-MET inhibitor (Ci), Gemcitabine (G) alone, in dual combinations (Hi+Ci, Hi+G, Ci+G), or in triple combination (Hi+Ci+G).  (B) The figure displays representative images of picrosirius red staining in mice that were either untreated (control) or treated with HGF inhibitor (Hi), c-MET inhibitor (Ci), Gemcitabine (G) alone, in dual combinations (Hi+Ci, Hi+G, Ci+G), or in triple combination (Hi+Ci+G).  (C) The graph displays quantitative data on α-smooth muscle actin staining in mice that were either vehicle-treated control or treated with the different drug combinations described in (A). All treatments did not affect α-smooth muscle actin staining compared to the control (p>0.05 vs. Control, n=7 to 9 animals/group).  (D) The graph shows quantitative data on picrosirius red staining for collagen in mice that were either vehicle-treated control or treated with the different drug combinations described in (C). All treatments did not affect picrosirius red staining compared to the control (p>0.05 vs. Control, n=7 to 9 animals/group).  Overall, the study examined the effects of different drug combinations on α-smooth muscle actin and collagen staining in mice, and the results suggest that none of the treatments had a significant effect on either staining compared to the control group.  (E) Representative images of Vimentin staining in untreated control mice and mice treated with HGF inhibitor (Hi), c-MET inhibitor (Ci), Gemcitabine (G) as single, dual (Hi+Ci, Hi+G, Ci+G), and triple combinations (Hi+Ci+G).  (F) Representative images of helper T-cell infiltration as assessed by CD4+ staining in untreated control mice and mice treated with HGF inhibitor (Hi), c-MET inhibitor (Ci), Gemcitabine (G) as single, dual (Hi+Ci, Hi+G, Ci+G) and triple combinations (Hi+Ci+G).  (G) Quantitative graph of Vimentin staining in vehicle-treated control mice and mice treated with HGF inhibitor (Hi), c-MET inhibitor (Ci), Gemcitabine (G) as single, dual (Hi+Ci, Hi+G, Ci+G) and triple combinations (Hi+Ci+G). Vimentin expression was unaffected by all treatments compared to control (p>0.05 vs. Control, n=7 to 9 animals/group).  (H) Quantitative graph of helper T-cell infiltration (CD4+ staining) in vehicle-treated control mice and mice treated with HGF inhibitor (Hi), c-MET inhibitor (Ci), Gemcitabine (G) as single, dual (Hi+Ci, Hi+G, Ci+G) and triple combinations (Hi+Ci+G). Helper T-cell infiltration was unaffected by all treatments compared to vehicle-treated (control) animals. (p>0.05 vs. Control, n=7 to 9 animals/group). |

| 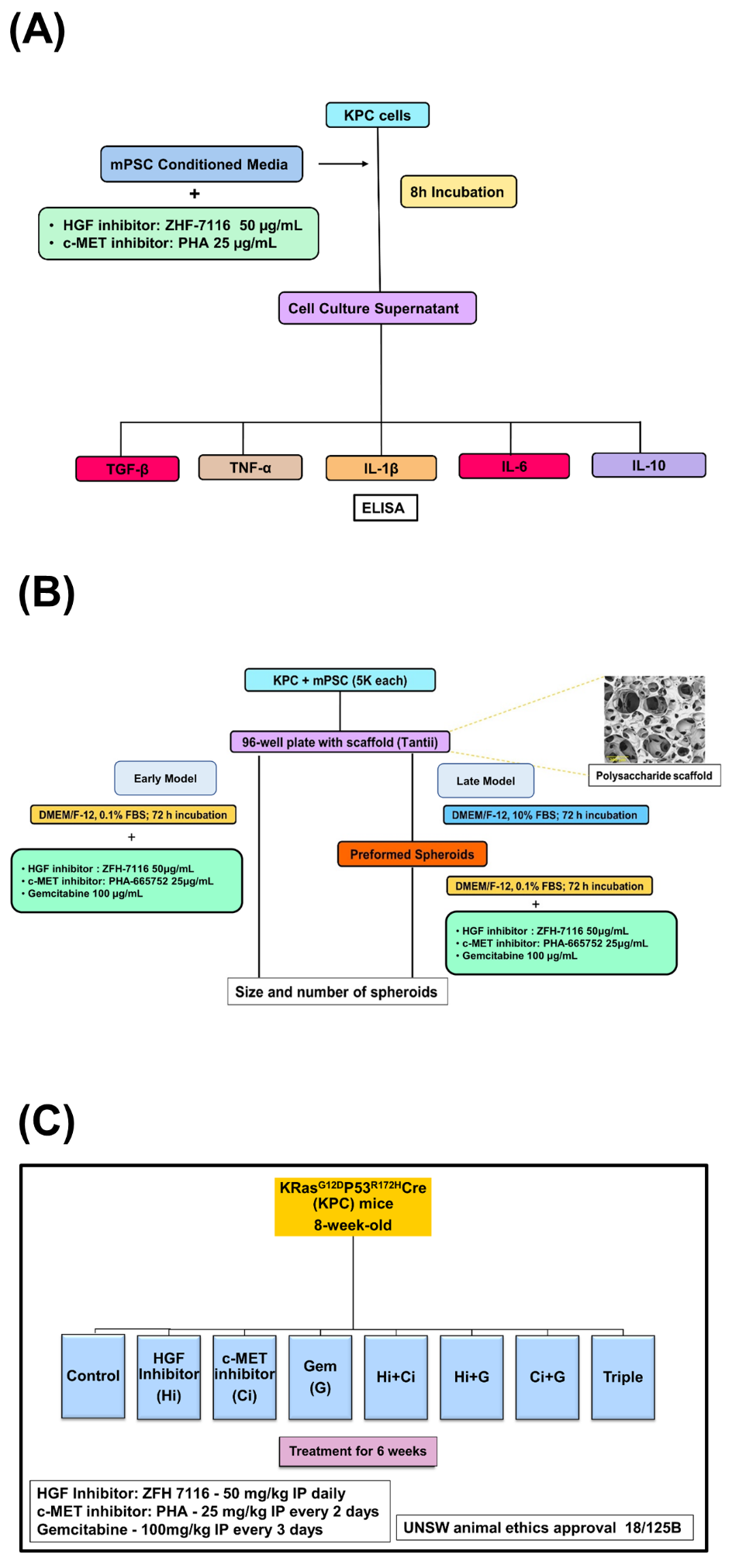 |
| --- |
| **Figure S7 Experimental design for in vitro cytokine production, 3D spheroid formation and genetically engineered mouse model**  (A) Scheme for evaluating the effect of HGF/c-MET inhibition on in vitro cytokines production by cancer cells  (B) Scheme for evaluating the effect of HGF/c-MET± gemcitabine on 3D spheroid formation  (C) Experimental design for the early intervention in genetically engineered mouse model |

| **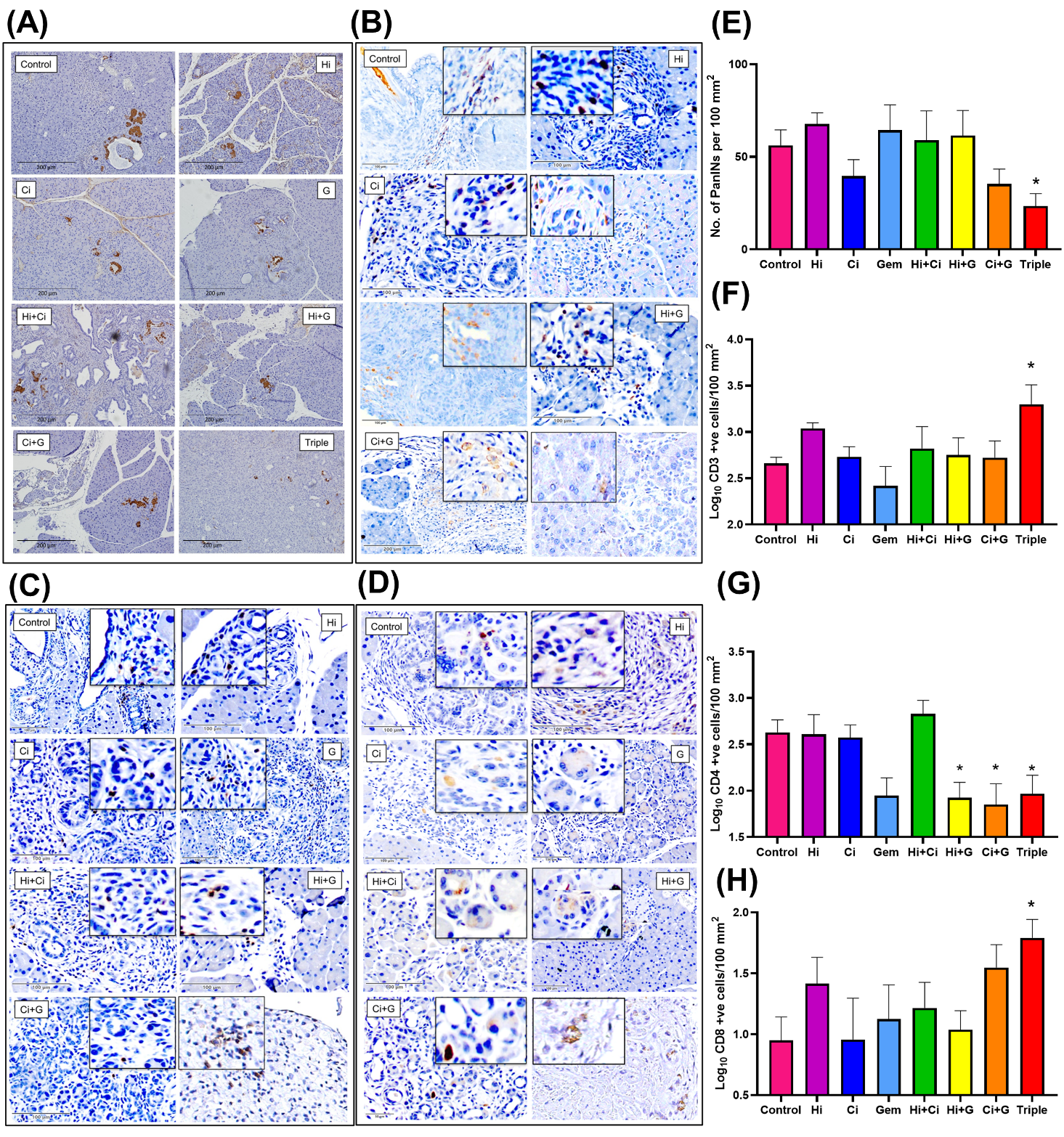** |
| --- |
| **Figure S8 HGF/c-MET pathway inhibition combined with gemcitabine decreases PanIN density and enhances T-cell infiltration in early genetically engineered KPC mouse model**  (A) Representative images of MUC5AC+ staining showing PanIN lesions in the pancreas of different treatment groups.  (B) Representative images of CD3+ staining showing total T-cell infiltration in the pancreas of different treatment groups.  (C) Representative images of CD4+ staining showing helper T-cell infiltration in the pancreas of different treatment groups.  (D) Representative images of CD8+ staining showing cytotoxic T-cell infiltration in the pancreas of different treatment groups.  (E) Quantitative graph showing PanIN density was significantly reduced by triple therapy compared to the Control. (*p<0.05 vs Control, n=6 animals/group).  (F) Quantitative graph showing total T-cell infiltration was significantly increased only in triple therapy compared to vehicle-treated (Control) animals. (*p<0.05 vs Control, n=6 animals/group).  (G) Quantitative graph showing helper T-cell infiltration was significantly decreased by gemcitabine in dual combination with HGF inhibitor or c-MET inhibitor and triple combination compared to vehicle-treated (Control) animals. (*p<0.05 vs. Control, n=6 animals/group).  (H) Quantitative graph showing cytotoxic T-cell infiltration was significantly increased by the dual combination of c-MET inhibitor and gemcitabine and triple therapy compared to vehicle-treated (Control) animals. (*p<0.05 vs. Control, n=6 animals/group). |

**Supplementary Tables**

| **Table S1 Median Inhibitory concentrations of ZFH7116 for various endogenous serine and cysteine proteases** | | |
| --- | --- | --- |
| **Target** | **IC_50_**  **(Molarity)** | **Endogenous Substrate** |
| HGFA | 23.00 | HGF |
| Matriptase | 14.00 | HGF, PAR-2, HAI-1 |
| Hepsin | 1.00 | HGF |
| TMPRSS2 | 74.00 | ACE2 |
| Factor Xa | 514.00 | Prothrombin |
| Thrombin | 7530.00 | Fibrinogen, Factor V, Factor VIII, PAR-1 |
| Cathepsin L | 7140.00 | Collagen, Elastin, Cathepsin B |
| Cathepsin S | 89.50 | Collagen |
| Cathepsin V | 10000.00 | Elastin |
| FVIIa | 2540.00 | Factor X |
| FXIa | 16.00 | Factor IX |
| Kallikrein 1 | 228.00 | Kininogen |
| Kallikrein 5 | 80.70 | High-molecular weight kininogen |
| Kallikrein 12 | 560.00 | Kininogen |
| Kallikrein 13 | 599.00 | Kininogen |
| Kallikrein 14 | 19.50 | Kininogen |
| Matriptase 2 | <1.02 | Unknown |
| Papain | 19700.00 | Unknown |
| Plasma Kallikrein | 2.34 | High-molecular weight kininogen |
| Plasmin | 134.00 | Fibrin |
| Proteinase A | 1880.00 | Unknown |
| Proteinase K | 5.19 | Unknown |
| Trypsin | <1.02 | Proteins |
| Tryptase b2 | <1.03 | PAR-2 |
| Tryptase g1 | <1.04 | Unknown |
| Urokinase | 7820.00 | Plasminogen |
| Calpain 1 | >20000 | Cytoskeletal proteins |
| Caspase 1 | >20000 | Interleukin-1beta |
| Caspase 2 | >20000 | Unknown |
| Caspase 3 | >20000 | Poly (ADP-ribose) polymerase, Cytoskeletal proteins |
| Caspase 4 | >20000 | Unknown |
| Caspase 5 | >20000 | Unknown |
| Caspase 6 | >20000 | Unknown |
| Caspase 7 | >20000 | Unknown |
| Caspase 8 | >20000 | FADD, cFLIP |
| Caspase 9 | >20000 | Apaf-1, Cytochrome c |
| Caspase 10 | >20000 | FADD |
| Caspase 11 | >20000 | Unknown |
| Caspase 14 | >20000 | Unknown |
| Cathepsin K | >20000 | Collagen, Elastin, Osteopontin |
| Chymase | >20000 | Unknown |
| Chymotrypsin | >20000 | Proteins |
| Elastase | >20000 | Elastin, Fibrinogen, Collagen |
| Kallikrein 7 | >20000 | Unknown |
| Cathepsin B | >20000 | Collagen, Elastin, Cathepsin L |
| Cathepsin C | >20000 | Unknown |
| Cathepsin H | >20000 | Unknown |
| Furin | >20000 | Proprotein convertases |

**Table S2 List of primary and secondary antibodies used for immunostaining the pancreatic sections**

| **Primary antibody** | **Antigen retrieval** | **Secondary antibody** | **DAB time** |
| --- | --- | --- | --- |
|  |  |  |  |
| **PanIN marker :**  Anti-mouse Mucin 5AC antibody, mouse monoclonal (AB3649, Abcam) (1.25 µg/mL) | 10 mM Citrate buffer pH 6.0 | Polyclonal anti-mouse HRP | 2 min |
| **Cancer cell marker:**  Anti-mouse pan-cytokeratin antibody, rabbit monoclonal (AB7753) (1 µg/mL) | 10 mM Citrate buffer pH 6.0 | Polyclonal anti-rabbit HRP | 2 min |
| **PSC – activation marker:**  Anti-mouse alpha-smooth muscle actin antibody, rabbit monoclonal (AB5694) (1:400) | 10 mM Citrate buffer pH 6.0 | Polyclonal anti-rabbit HRP | 2 min |
| **Stemness marker:**  Anti-mouse DCLK1 antibody, rabbit monoclonal (AB31704) (1:100) | 10 mM Citrate buffer pH 6.0 | Polyclonal anti-rabbit HRP | 2 min |
| **T-cell subset markers:**  Anti-mouse CD3 antibody, rabbit monoclonal (AB16669) (1:150)  Anti-mouse CD4 antibody, rabbit monoclonal (AB183685) (1:1000)  Anti-mouse CD8 antibody, rabbit monoclonal (AB237723) (1:800) | 10 mM Tris-EDTA buffer pH 9.0 | Polyclonal anti-rabbit HRP | 9 min |
| **EMT markers** :  Anti-mouse E-cadherin antibody, rabbit monoclonal (AB231303) (1:100)  Anti-mouse Vimentin antibody, rabbit monoclonal (AB8978) (1:100) | 10 mM Citrate buffer pH 6.0 | Polyclonal anti-rabbit HRP | 2 min |
| **M2-type macrophages**  Anti-mannose (CD206) antibody, rabbit polyclonal (AB64693) (0.1µg/mL) | 10 mM Tris-EDTA buffer pH 9.0 | Polyclonal anti-rabbit HRP | 9 min |
| **Natural Killer cells**  Anti-NK 1.1 antibody, mouse monoclonal (MA1-70100) Invitrogen | 10 mM Tris-EDTA buffer pH 9.0 | Polyclonal anti-rabbit HRP | 9 min |
| **Myeloid derived suppressor cells** Anti-CD33 antibody, rabbit polyclonal (AB203032) (1:100) | 10 mM Tris-EDTA buffer pH 9.0 | Polyclonal anti-rabbit HRP | 9 min |
|  |  |  |  |

**Table S3 The limit of detection of the ELISA for cytokine analysis**

| **Cytokine** | **Sensitivity (pg/mL)** | **Range (pg/mL)** | **Catalogue Number** |
| --- | --- | --- | --- |
| Mouse TGF- beta | 7.8 | 31.30 – 2000 | AB119557 |
| Mouse TNF-alpha | 9.1 | 46.88 – 3000 | AB208348 |
| Mouse IL-1 beta | 1.0 | 1.56 – 100 | AB197742 |
| Mouse IL-6 | <2.0 | 0.82 – 600 | AB100712 |
| Mouse IL-10 | <45.0 | 78.13 – 5000 | AB100697 |

**List of abbreviations**

16S rRNA - 16S ribosomal RNA

3D - Three-dimensional

ACEC - Animal Care and Ethics Committee

ADONIS - Analysis of similarity

Ci - c-MET inhibitor

CM - Conditioned medium

c-MET - Mesenchymal-epithelial transition factor

DMEM - Dulbecco's Modified Eagle's Medium

DMSO - Dimethyl Sulfoxide

EMT - Epithelial-mesenchymal transition

FBS - Foetal Bovine Serum

FOLFIRNOX - Combination chemotherapy regimen (folinic acid, fluorouracil, irinotecan, and oxaliplatin)

Gem - Gemcitabine

H&E - Hematoxylin and Eosin

HGF - Hepatocyte growth factor

Hi - HGF inhibitor

IMDM - Iscove's Modified Dulbecco's Medium

KPC - KrasLSL-G12D/+, Trp53LSL-R172H, Pdx1-cre

K-rasLA1 - Mouse model of pancreatic cancer with a K-ras mutation

MAPK - Mitogen-activated protein kinase

mPSC - Mouse pancreatic stellate cells

Nab-paclitaxel - Nanoparticle albumin-bound paclitaxel, another chemotherapy drug used for pancreatic cancer

OTUs - Operational taxonomic units

PC - Pancreatic cancer

PCoA - Principal coordinates analysis

PHA665752 - c-Met inhibitor compound

PI3K - Phosphoinositide 3-kinase

PSCs - Pancreatic Stellate Cells

RNA - Ribonucleic acid

TGF-β - Transforming Growth Factor-beta

USA - United States of America

ZFH-7116 - HGF inhibitor compound
